# Biofunctional lipid nanoparticles for precision treatment and prophylaxis of bacterial infections

**DOI:** 10.1126/sciadv.adk9754

**Published:** 2024-04-05

**Authors:** Xinran Peng, Jiaoyu Chen, Yingying Gan, Li Yang, Yuanjing Luo, Changxin Bu, Yi Huang, Xinhai Chen, Jeremy Tan, Yi Yan Yang, Peiyan Yuan, Xin Ding

**Affiliations:** ^1^ School of Pharmaceutical Sciences (Shenzhen), Shenzhen Campus of Sun Yat-sen University, Shenzhen 518107, PR China.; ^2^ Institute of Infectious Diseases, Shenzhen Bay Laboratory, Shenzhen 518132, PR China.; ^3^ Bioprocessing Technology Institute, Agency for Science, Technology and Research (A*STAR), 20 Biopolis Way, Centros #06-01, Singapore 138668, Singapore.; ^4^ School of Medicine, Shenzhen Campus of Sun Yat-sen University, Shenzhen 518107, PR China.; ^5^ State Key Laboratory of Anti-Infective Drug Discovery and Development; School of Pharmaceutical Sciences, Sun Yat-sen University, Guangzhou 510006, China.

## Abstract

The lack of bacterial-targeting function in antibiotics and their prophylactic usage have caused overuse of antibiotics, which lead to antibiotic resistance and inevitable long-term toxicity. To overcome these issues, we develop neutrophil-bacterial hybrid cell membrane vesicle (HMV)–coated biofunctional lipid nanoparticles (LNP@HMVs), which are designed to transport antibiotics specifically to bacterial cells at the infection site for the effective treatment and prophylaxis of bacterial infection. The dual targeting ability of HMVs to inflammatory vascular endothelial cells and homologous Gram-negative bacterial cells results in targeted accumulation of LNP@HMVs in the site of infections. LNP@HMVs loaded with the antibiotic norfloxacin not only exhibit enhanced activity against planktonic bacteria and bacterial biofilms in vitro but also achieve potent therapeutic efficacy in treating both systemic infection and lung infection. Furthermore, LNP@HMVs trigger the activation of specific humoral and cellular immunity to prevent bacterial infection. Together, LNP@HMVs provide a promising strategy to effectively treat and prevent bacterial infection.

## Teaser

Hybrid membrane–coated lipid nanoparticles act as a dual-targeted delivery vehicle of antibiotics and an antibacterial vaccine.

## INTRODUCTION

Antibiotics are the main treatment option for bacterial infections, and most antibiotics kill bacteria by interfering with essential processes after entering the bacterial cells (*1*). However, antibiotics are subjected to blood clearance in the systemic circulation, and physical barriers of bacterial cell wall and membrane hinder the penetration of antibiotics, leading to low accumulation of antibiotics inside bacterial cells. Moreover, the uptake of antibiotics by bacteria embedded in biofilm is further greatly reduced because of the presence of extracellular polymeric substances in the biofilm. Therefore, intensive and multiple doses of antibiotics are usually needed to achieve desired therapeutic effect (*2*–*4*). The repeated use of most antibiotics induces inevitable toxicity to normal tissues and organs, and promotes the emergence of drug-resistant bacteria, which deceases the efficacy of antibiotics (*5*, *6*). Another common cause for antibiotic overuse is prophylactic usage to prevent the occurrence of bacterial infections, especially to prevent post-surgery infections (*7*, *8*). Unfortunately, the antibiotic prophylaxis is the only clinically available option to prevent the occurrence of most bacterial infections after surgery, as there are no effective vaccines available for the most prevalent bacterial pathogens such as *Escherichia coli* and *Staphylococcus aureus* (*9*, *10*). Therefore, a nanotechnology-based strategy, which is able to simultaneously deliver antibiotics into bacterial cells and activate the immune system to prevent occurrence of infections, would be ideal to address clinical challenges of antibiotic usage, including unsatisfied therapeutic effect, long-term toxicity, and induction of antibiotic resistance.

Bacterial outer membrane vesicles (OMVs) are nanoparticles naturally secreted by Gram-negative bacteria through outward budding, thus having similar structure and composition as bacterial outer membrane does (*11*, *12*). Since OMVs can act as communication tools among bacteria cells and are readily fusible with bacteria (*13*), Huang and our team have used OMVs or OMV-coated nanoparticles as antibiotic carriers to achieve intracellular delivery and enhance antibacterial activity at lower doses (*14*, *15*). In addition, homologous bacterial targeting property of OMVs was also proven. However, OMV delivery systems are not suitable for treatment of systemic infection or administered through systemic injection, as they lack active targeting function to infection site and are easily recognized and cleared by the immune system due to their surface antigens. The bacterial antigens of OMVs can recognize and activate the immune system, and therefore, OMVs have been used as vaccines (*16*). Several OMVs such as OMVs derived from *Neisseria meningitidis serogroup B * have been clinically used as antibacterial vaccines (*17*), while other OMV-based vaccines derived from *E. coli*, *Pseudomonas aeruginosa*, *Klebsiella pneumoniae*, and other pathogens are still under preclinical investigation (*18*–*20*).

**Fig. 1.**
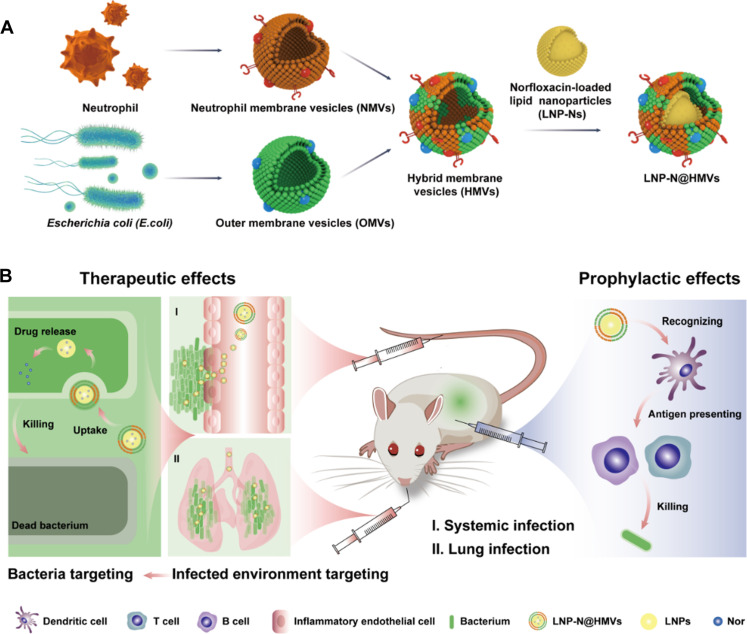
Schematic illustrations of the construction and application of LNP-N@HMVs. (**A**) Construction of hybrid cell membrane–coated antibiotic delivery system LNP-N@HMVs. (**B**) Application of LNP-N@HMVs in dual-targeted treatment and prophylaxis of bacterial infections.

In this study, we designed antibiotic-loaded biofunctional lipid nanoparticles (LNPs) and the surface of the nanoparticles was coated with both OMVs and neutrophil membrane vesicles (NMVs) for transporting the antibiotic to inflammatory microenvironment and bacterial cells, and for prophylaxis of bacterial infections (Fig. 1). Neutrophil is one of the most abundant white blood cells, and it has been proven that NMVs are able to target inflammatory tissue and escape immune elimination (*21*–*24*). To overcome the nonspecific issue of OMVs as drug delivery vehicles, NMVs were hybridized with *E. coli*–derived OMVs to prepare hybrid cell membrane vesicles (HMVs) for targeting the inflammatory microenvironment. The HMV-coated LNPs (LNP@HMVs) were loaded with the antibiotic fluoroquinolone norfloxacin (Nor) to form a therapeutic nanoparticulate system (LNP-N@HMVs) for enhancing the transportation and uptake of antibiotics by bacteria in vivo. Furthermore, because of the immune activation ability of OMVs, LNP@HMVs were also leveraged to activate the specific humoral and cellular immunity as an antibacterial vaccine to prevent infections. To verify our hypothesis, we investigated the targeted uptake of LNP@HMVs by inflammatory endothelial cells and Gram-negative bacteria in vitro, and their targeted accumulation at the bacterial infection site in vivo. Antibacterial activity of LNP-N@HMVs against Gram-negative bacteria *E. coli*, *K. pneumoniae*, and *E. coli* biofilm in vitro, and therapeutic efficacy in an *E. coli*–induced mouse peritonitis model (systemic infection) and a *K. pneumoniae*–induced mouse lung infection model (local infection) were studied. Moreover, the in vivo activation of T cell and B cell immunity and prophylaxis for peritonitis was evaluated.

## RESULTS

### Preparation and characterization of LNP-N@HMVs

**Fig. 2.**
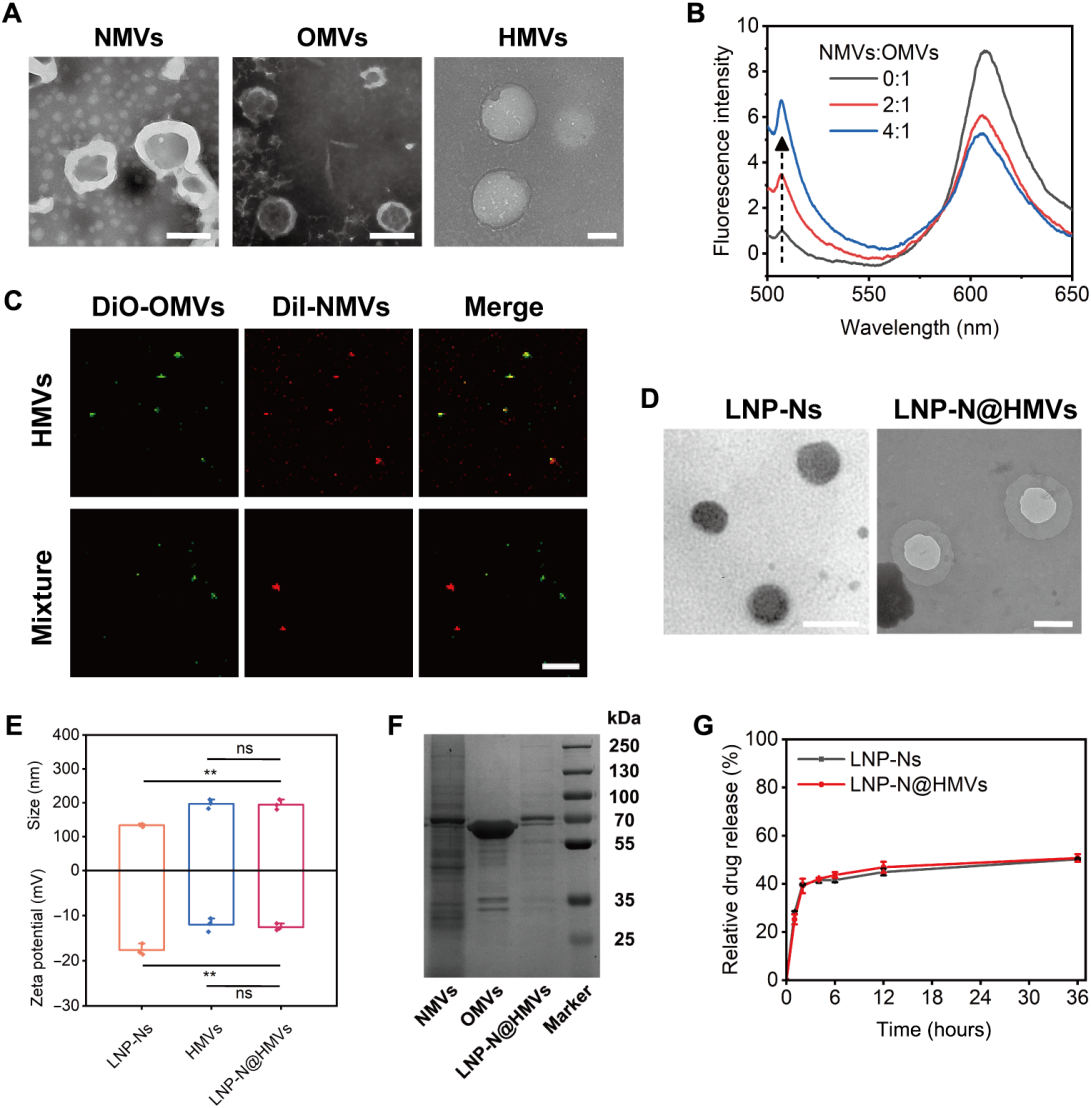
Characterization of HMVs and LNP-N@HMVs. (**A**) TEM images of *E. coli*–derived OMVs, HL-60 cell–derived NMVs, and HMVs. Scale bars, 200 nm. (**B**) Fluorescent spectra of OMVs labeled with FRET dye pair (DiO and DiI) after sonication with unlabeled NMVs at different protein weight ratios. (**C**) Fluorescence colocalization images of HMVs and a mixture of DiO-labeled OMVs and DiI-labeled NMVs. Scale bars, 5 μm. (**D**) TEM images of LNP-Ns and LNP-N@HMVs. Scale bars, 200 nm. (**E**) Hydrodynamic size and zeta potential of LNP-Ns, HMVs, and LNP-N@HMVs. *n* = 3; **P < 0.01; ns, nonsignificance. (**F**) SDS-PAGE protein analysis of NMVs, OMVs, and LNP-N@HMVs. (**G**) Nor release profiles from LNP-Ns and LNP-N@HMVs at pH 7.4 over 36 hours (*n* = 3).

NMVs and OMVs were isolated from immortalized neutrophil-like cell line (HL-60) and model Gram-negative bacteria *E. coli*, respectively (*15*, *25*, *26*). Both NMVs and OMVs exhibited typical exosome morphology (Fig. 2A). NMVs and OMVs were subsequently fused by sonication and extrusion at a 1:1 protein weight ratio to form HMVs with size of about 200 nm in diameter (Fig. 2A). To verify the fusion process of NMVs and OMVs, Förster resonance energy transfer (FRET) assay was performed. A FRET dye pair [1,1′-dioctadecyl-3,3,3′,3′-tetramethylindocarbocyanine perchlorate (DiI) and 3,3′-dioctadecyloxacarbocyanineperchlorate (DiO)] was used to label the OMVs, followed by sonication with unlabeled NMVs at different protein weight ratios (Fig. 2B). With increasing amounts of NMVs, enhanced fluorescence emission of DiO and reduced fluorescence emission of DiI were observed because of the enlarged distance of the dyes in OMVs after hybridization with NMVs, indicating successful fusion of OMVs and NMVs. The fusion of OMVs and NMVs after sonication and extrusion was further confirmed by the fluorescence overlap (yellow) of DiI-labeled NMVs (red) and DiO-labeled OMVs (green) (Fig. 2C), while the fluorescence of NMVs and OMVs stays separated if they were simply mixed without any treatment.

The HMVs were subsequently coated on Nor-loaded LNPs (LNP-Ns) through sonication and extrusion, where LNP-Ns were fabricated by a solvent diffusion method with glyceryl monostearate (GMS) and Nor in organic phase (weight ratio of GMS to Nor = 7:1) and poloxamer188 in aqueous phase (table S1). LNP-N@HMVs exhibited a spherical core-shell morphology (Fig. 2D). Both a larger size compared to LNP-Ns (Fig. 2E) and FRET assay (fig. S1) further confirmed that the HMVs were coated on the surface of LNPs instead of forming a fused hybrid membrane vesicle. LNP-N@HMVs inherited most of cell membrane proteins of NMVs and OMVs, and thus, the functionality of NMVs and OMVs could remain in the LNP-N@HMVs (Fig. 2F). The release profiles of Nor from LNP-Ns and LNP-N@HMVs were measured, and approximately 25 and 50% of the drug were released in 1 and 36 hours, respectively (Fig. 2G). The presence of HMV did not notably affect the release profile of Nor.

### In vitro targeting of LNP@HMVs to inflammatory endothelial cells and bacteria

**Fig. 3.**
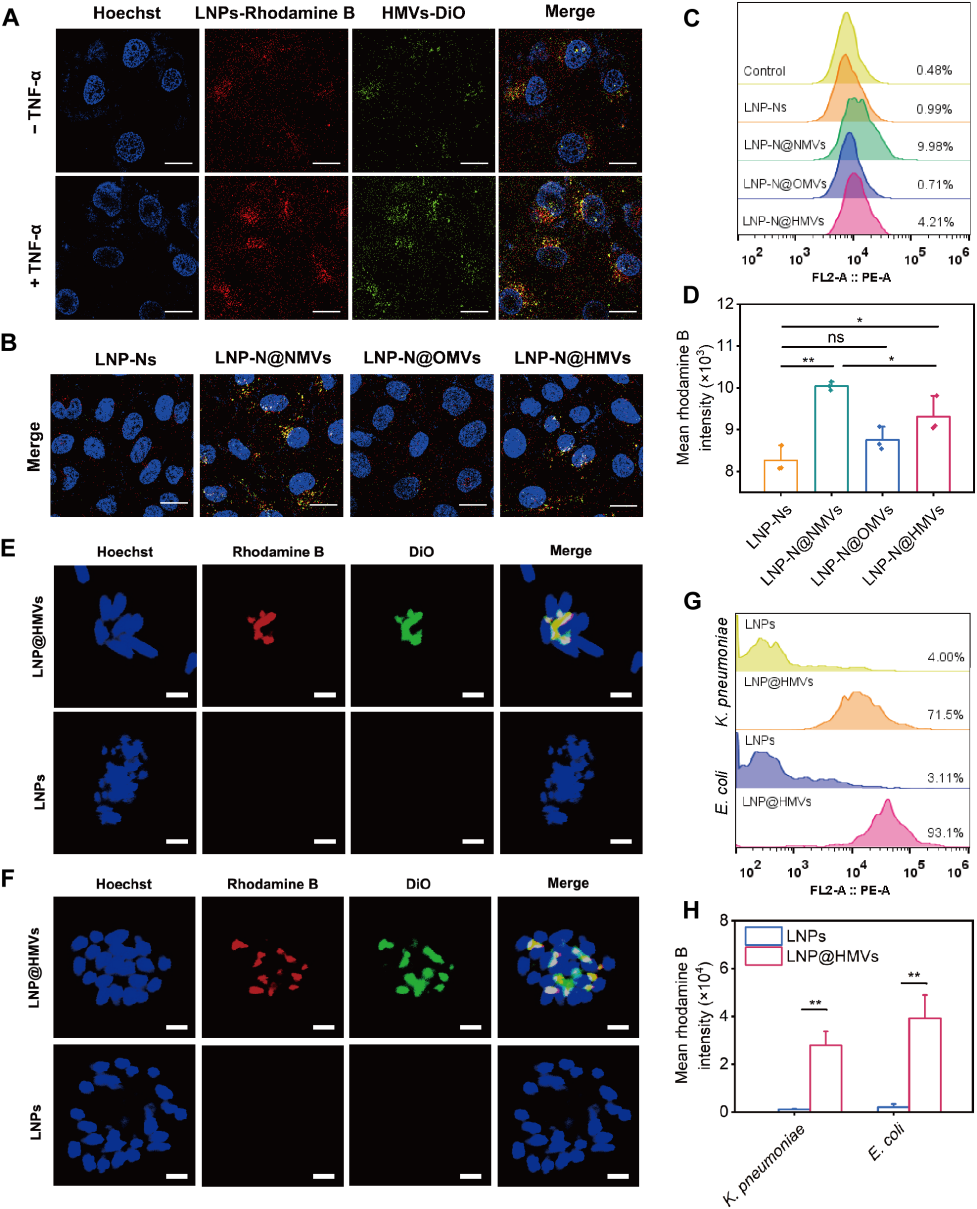
In vitro targeting of LNP@HMVs to inflammatory endothelium cells and homologous bacteria. (**A**) Confocal microscopic images of nonactivated or TNF-α–activated HUVECs cultured with LNP-N@HMVs (concentration, 20 μg ml^−1^ NPs) for 0.5 hour, where rhodamine B (red) and DiO (green) were used to stain LNPs and HMVs, respectively. Scale bars, 20 μm. (**B**) Confocal microscopic images and (**C** and **D**) flow cytometry analysis of TNF-α–activated HUVECs cultured with LNP-Ns, LNP-N@NMVs, LNP-N@OMVs, or LNP-N@HMVs for 1 hour (concentration, 20 μg ml^−1^ NPs). Scale bars, 20 μm. Confocal microscopic images of (**E**) *E. coli* and (**F**) *K. pneumoniae* and (**G** and **H**) flow cytometry analysis of rhodamine B in these bacteria after incubation with LNPs or LNP@HMVs for 1 hour (concentration, 20 μg ml^−1^ NPs). Scale bars, 2 μm. *n* = 3; *P < 0.05, **P < 0.01.

NMVs derived from differentiated neutrophil-like HL-60 cells inherited their binding ability to inflammatory endothelial cells in vitro (*27*). To verify whether LNP@HMVs inherited this property from NMVs, LNP-N@HMVs (rhodamine B–labeled LNPs, DiO-labeled HMVs) were incubated with human umbilical vein cells (HUVECs). Confocal laser scanning microscopic (CLSM) images showed stronger colocalized fluorescent signal (yellow) of rhodamine B and DiO in tumor necrosis factor–α (TNF-α)–activated HUVECs than normal HUVECs, indicating targeting ability of LNP-N@HMVs to inflammatory vascular cells (Fig. 3A). Consistent with the CLSM results, significantly higher mean fluorescence intensity of rhodamine B and DiO in the TNF-α–activated HUVECs was also observed in flow cytometry (fig. S2). The targeting property of LNP-N@HMV to inflammatory vascular cells is due to the specific binding of β2 integrin expressed on NMVs and ICAM-1 (intercellular cell adhesion molecule–1) presented on inflammatory vascular endothelial cells (*27*). Owing to the targeting property of NMVs, the nanoparticles coated with NMVs (LNP-N@NMVs) showed the strongest fluorescence in TNF-α–activated cells, followed by hybrid membrane–coated nanoparticles (LNP-N@HMVs) (Fig. 3, B to D, and figs. S3 and S4). Meanwhile, the fluorescence of LNP-N@OMVs in HUVECs was not significantly different as compared to nonfunctionalized LNP-Ns, and the fluorescence of LNP-Ns in HUVECs was similar to the cells without any treatment (Fig. 3D). Therefore, the coating of NMVs or HMVs is necessary to enhance the specific binding of LNPs to the inflammatory cells. As the LNP-N@HMVs need to traverse the inflammatory vascular endothelial cells before reaching the bacterial pathogens, we conducted transwell assay to investigate whether the LNP-N@HMVs can penetrate the endothelial cell layers (fig. S5). We observed a higher fluorescence intensity of LNP-N@HMVs, compared to that of LNP-Ns, in the bottom chamber, indicating the ability of HMVs to target inflammatory vascular cells and traverse them. In addition, a stronger fluorescence signal was noted in the TNF-α–activated HUVEC group compared to the normal HUVEC group, further confirming the enhanced efficacy of LNP-N@HMVs in targeting inflammatory vascular cells.

The uptake of antibiotics such as Nor in bacterial cells is crucial for the antibacterial activity, as antibiotics need to interact with intracellular target for bacterial inhibition or killing. *E. coli*–derived OMVs are favorably internalized by *E. coli* and other Gram-negative bacteria such as *K. pneumoniae*, which is due to the similar composition and structure between OMVs and bacterial membranes (fig. S6). The LNP@HMVs also inherited this bacteria-targeting property from *E. coli*–derived OMVs, which was evidenced by the strong fluorescence signal of LNP@HMVs in Gram-negative bacteria (*E. coli* and *K. pneumoniae*) and much less fluorescence signal in Gram-positive bacteria (*S. aureus*) as characterized by CLSM and flow cytometry (Fig. 3, E to H, and figs. S7 and S8). Meanwhile, the LNPs without membrane coating hardly penetrated through the Gram-negative bacterial cell wall/membrane, as negligible fluorescence of LNPs was observed (Fig. 3, E to H). The distinct difference in bacterial cellular uptake of LNPs and LNP@HMVs demonstrates that HMVs endowed LNPs with the homotypic targeting function to Gram-negative bacteria, implying that LNP@HMVs could selectively enter and deliver the encapsulated antibiotic to the specific bacteria.

### In vitro antibacterial and antibiofilm activity of LNP-N@HMVs

**Fig. 4.**
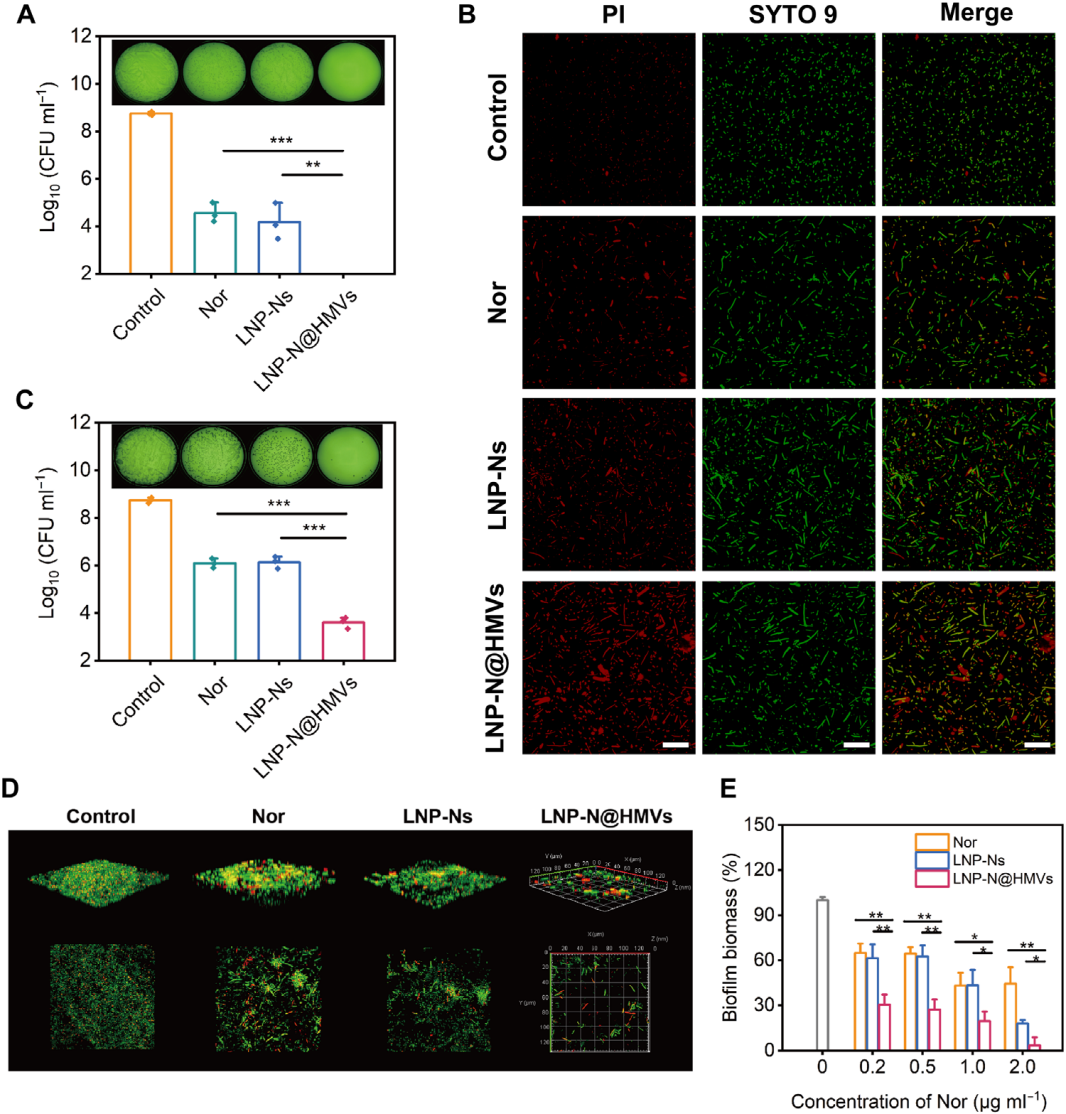
In vitro antibacterial and antibiofilm activity of LNP-N@HMVs. (**A**) Photographs and quantification of the bacterial colonies after treating *E. coli* (~10^5^ CFU ml^−1^) with Nor, LNP-Ns, or LNP-N@HMVs with equivalent Nor concentration at 0.12 μg ml^−1^ for 8 hours. Bacteria without any treatment were used as control. Limit of detection: 100 CFU ml^−1^. (**B**) Confocal microscopic images of *E. coli* (~10^8^ CFU ml^−1^) with different treatments at the equivalent Nor concentration of 0.24 μg ml^−1^ and stained with Live/Dead BacLight bacterial viability kit (green, all the bacteria; red, dead bacteria). Scale bar, 20 μm. (**C**) Photographs and quantification of the colonies of *K. pneumoniae* after the same treatments as that of *E. coli* (A). Limit of detection: 100 CFU ml^−1^. (**D**) Confocal microscopic images of *E. coli* biofilm with different treatments at the equivalent Nor concentration of 0.5 μg ml^−1^ for 24 hours and stained with Live/Dead BacLight bacterial viability kit. (**E**) Biomass of *E. coli* biofilm after treatment with different formulations at various Nor concentrations. *n* = 3; *P < 0.05, **P < 0.01, and ***P < 0.001.

HMV coating has been proven to enhance the uptake of LNPs by homologous bacteria and bacteria with similar cell membrane structure, which could be beneficial for the targeted delivery of Nor using LNP-N@HMVs and strengthening the antibacterial effect of Nor. To verify this hypothesis, bacterial killing efficacy and Live/Dead cell staining assay against *E. coli* were performed to evaluate antibacterial activity (Fig. 4, A and B). Compared with free antibiotic Nor and LNPs loaded with Nor (LNP-Ns), LNP-N@HMVs exhibited higher antibacterial potency and eliminated all the bacteria with equivalent Nor concentration at 0.12 μg ml^−1^. Meanwhile, the Nor or LNP-Ns showed mild antibacterial activity with approximately 1-log reduction of colony-forming units (CFUs) compared with initial bacterial loading (Fig. 4A), and a great number of live *E. coli* (green) were observed (Fig. 4B). Notably, HMV coating improved antibacterial activity of the encapsulated Nor toward not only homologous bacteria *E. coli* but also other Gram-negative bacteria with similar cell membrane structure such as *K. pneumoniae*, which is a common pathogen responsible for lung infections. LNP-N@HMVs caused significant CFU reduction of *K. pneumoniae*, while Nor or LNP-Ns at the same equivalent Nor concentration were not capable of killing bacteria but only delaying their growth (Fig. 4C). These results indicated that HMV coating significantly enhanced antibacterial efficacy of LNP-Ns toward Gram-negative bacteria due to the enhanced intracellular uptake of the antibiotic.

Because of the protective effects of extracellular polymeric substances in biofilms, the killing of bacteria in the established biofilms is typically more difficult than that of planktonic bacteria (*28*, *29*). As OMVs play an important role in biofilm formation and cell-cell communication, and biofilms are also the main reservoirs of OMVs (*30*, *31*), we postulated that the components of OMVs in HMVs can enhance the penetration of LNP-N@HMVs in biofilm and thus improve antibacterial activity against bacteria in biofilm. This hypothesis was verified by treating the established *E. coli* biofilm with LNP-N@HMVs. The Live/Dead bacterial staining showed that a dense *E. coli* biofilm was formed after incubation for 48 hours (Fig. 4D). Although free Nor and LNP-Ns removed a small portion of bacterial biofilm, large bacterial aggregates were still observed. In sharp contrast, LNP-N@HMVs with the equivalent Nor concentration at 0.5 μg ml^−1^ nearly removed all the bacteria in the biofilm. The amount of biomass in the biofilm after treatment characterized by crystal violet staining also showed the same trend as CLSM (Fig. 4E). The antibiofilm activity of LNP-N@HMVs was further improved by increasing the concentration. Most of the biomass was eradicated by LNP-N@HMVs with the equivalent Nor concentration at 2.0 μg ml^−1^. The enhanced antibiofilm activity of LNP-N@HMVs was probably due to the penetration and mobility of OMVs in biofilm and the enhanced uptake of Nor by bacteria.

### In vitro and in vivo biocompatibility of LNP-N@HMVs

In vitro and in vivo biocompatibility of LNP-N@HMVs was evaluated before the study of in vivo treatment efficacy toward bacterial infections. The viability of HUVECs after treatment with LNP@HMVs remained ~95% even with the concentration of LNP@HMVs reaching 160 μg ml^−1^ (fig. S9). This finding indicated that LNP@HMVs were not toxic to mammalian cells. The toxicity of LNP-N@HMVs in vivo was evaluated by blood analysis. LNP-N@HMVs with equivalent Nor dose at 25 μg/20 g mouse body weight, which were used for the following in vivo antibacterial efficacy evaluation, were injected intravenously to male C57BL/6 mice. After 7 days, there was no mortality in all groups, and blood analysis was performed. All the biochemical parameters including alkaline phosphatase (ALT), aspartate aminotransferase (AST), blood urea nitrogen (UREA), creatinine (CREA), uric acid (UA), potassium (K), and sodium (Na) in LNP-N@HMV–treated mice were similar to the phosphate-buffered saline (PBS)–treated mice, and no significant difference (*P* > 0.05) was noted (table S2). Moreover, blood cell counts including white blood cells, lymphocytes, monocytes, neutrophils, red blood cells, and platelets of the LNP-N@HMV–treated mice and PBS-treated mice also showed no significant difference (*P* > 0.05) (table S3). The blood analysis further proved the biosafety of LNP-N@HMVs. This is due to the low amount of Nor used and the biocompatibility of LNP@HMVs. In addition, although the endotoxin, which is one of the virulent factors that could provoke systemic inflammatory responses (*32*), is also present in OMVs, the content of endotoxin in OMVs was determined to approximately 20 times lower than that in bacterial outer membrane [2.2 versus 47.8 endotoxin unit (EU) ng^−1^] (table S4). The greatly reduced amount of endotoxin suggested that OMVs were much safer than bacterial membrane and contributed to the nontoxicity of LNP-N@HMVs.

### In vivo targeting and therapeutic effects of LNP-N@HMVs

**Fig. 5.**
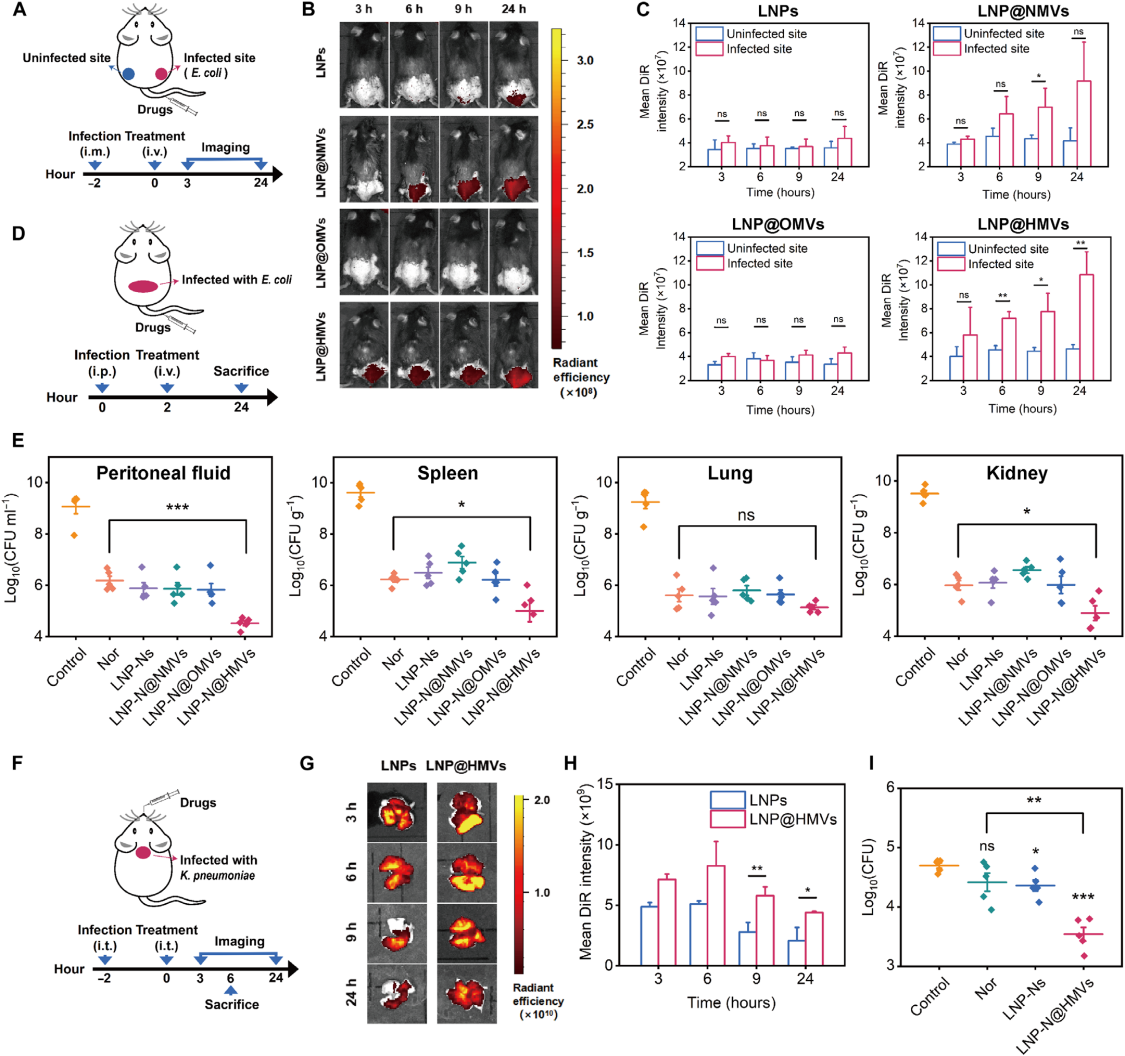
In vivo targeting and therapeutic effects of LNP-N@HMVs against bacterial infections. (**A**) Schematics of the experiments to evaluate targeting function of LNP@HMVs in a mouse muscle infection model. (**B**) Representative fluorescence images of the infected mice under different treatments at 3, 6, 9, and 24 hours after injection. (**C**) Mean DiR fluorescence intensity of the infected and the uninfected sites in different treatment groups (*n* = 3). (**D**) Schematics of the experiments to evaluate the therapeutic efficacy of LNP-N@HMVs (equivalent Nor dose, 25 μg/20 g) in treating mice with *E. coli*–induced peritonitis. (**E**) Quantification of the number of bacteria in peritoneal fluid, spleen, lung, and kidney of the infected mice with different treatments. Infected mice without any treatment were used as control (*n* = 5). (**F**) Schematics of the experiments to evaluate targeting and therapeutic efficacy of LNP-N@HMVs (equivalent Nor dose, 1 μg/20 g) in treating mice with *K. pneumoniae*–induced lung infection. (**G**) Representative fluorescence images and (**H**) mean DiR fluorescence intensity of the infected mice under different treatments (*n* = 3). (**I**) Quantification of the number of bacteria in the infected lung tissues of mice under different treatments (*n* = 5). *P < 0.05, **P < 0.01, and ***P < 0.0001.

As LNP@HMVs could effectively target to inflammatory vascular cells and bacteria in vitro, the in vivo targeted accumulation of LNP@HMVs at infection site was further investigated in a muscle infection mouse model induced by *E. coli* (Fig. 5A). After tail vein injection of LNPs, LNPS@NMVs, LNPs@OMVs, or LNP@HMVs at 2 hours after infection, the strong fluorescence signals of LNPs@NMVs and LNPs@HMVs were observed at 24 hours after injection, while almost no fluorescence signal of LNPs and LNPs@OMVs was found at the infection site (Fig. 5B). These findings suggested that LNPs and OMV-coated LNPs did not show targeted accumulation probably due to the OMV-triggered immune clearance in the blood circulation. Notably, the fluorescence signals from both LNP@NMVs and LNP@HMVs were found at the infected site at 3 to 24 hours after injection, and the fluorescence signals of the infected site gradually increased over time likely with the progression of infection (Fig. 5C), confirming the infection site–targeting property of NMVs and HMVs. The difference in the targeted accumulation between LNP@HMVs and LNP@OMVs suggested that the fusion with NMVs to form HMVs can overcome the immune clearance problem of OMVs and enabled effective dual targeting to infected microenvironment and further to the bacteria. This property is beneficial for the efficient delivery of antibiotics from the circulation system to the bacteria at the infection site.

To investigate the therapeutic effects of LNP-N@HMVs in vivo, a systemic infection model (*E. coli*–induced peritonitis) and a *K. pneumoniae*–induced lung infection model were established. In the peritonitis model, although the bacterial count in the peritoneal fluid of the infected mice treated with free Nor, LNP-Ns, LNP-N@NMVs, or LNP-N@OMVs (equivalent Nor dose: 25 μg/20 g) all decreased compared with the infected mice without any treatment, LNP-N@HMVs exhibited the highest antibacterial potency with greater bacterial reduction than free Nor (Fig. 5, D and E). Meanwhile, the in vivo antibacterial efficacy of LNP-Ns, LNP-N@NMVs, and LNP-N@OMVs was similar to that of free Nor, indicating the necessity of HMV functionalization for enhancement of antibacterial potency, as the encapsulation with LNPs and functionalization with mono-membrane OMVs or NMVs were not able to improve antibacterial activity of Nor. In addition, LNP-N@HMVs also exhibited significantly higher *E. coli* elimination efficacy especially in the spleen and kidney than free Nor, showing great potential for the treatment of peritonitis-induced systemic infection (Fig. 5E). Apart from excellent therapeutic efficacy for the treatment of the systemic infection, LNP-N@HMVs were also able to treat *K. pneumoniae*–induced lung infection through intratracheal administration (Fig. 5F). Compared with LNPs, LNP@HMVs displayed higher accumulation and longer retention time in the infected lung tissue (Fig. 5G), and significant difference in the fluorescence intensity between LNPs and LNP@HMVs was observed at 9 and 24 hours after injection (Fig. 5H). The targeted accumulation of LNP-N@HMVs led to high therapeutic efficacy with over 90% bacterial reduction after only one-dose treatment, while the free antibiotic treatment was not able to reduce bacterial load in the infected lungs (Fig. 5I). The enhanced therapeutic efficacy against both peritonitis and pneumonia models demonstrated the great potential of LNP-N@HMVs for dual-targeted delivery of antibiotics and treatment of Gram-negative bacterial infections.

### In vitro and in vivo immune responses activated by LNP@HMVs

**Fig. 6.**
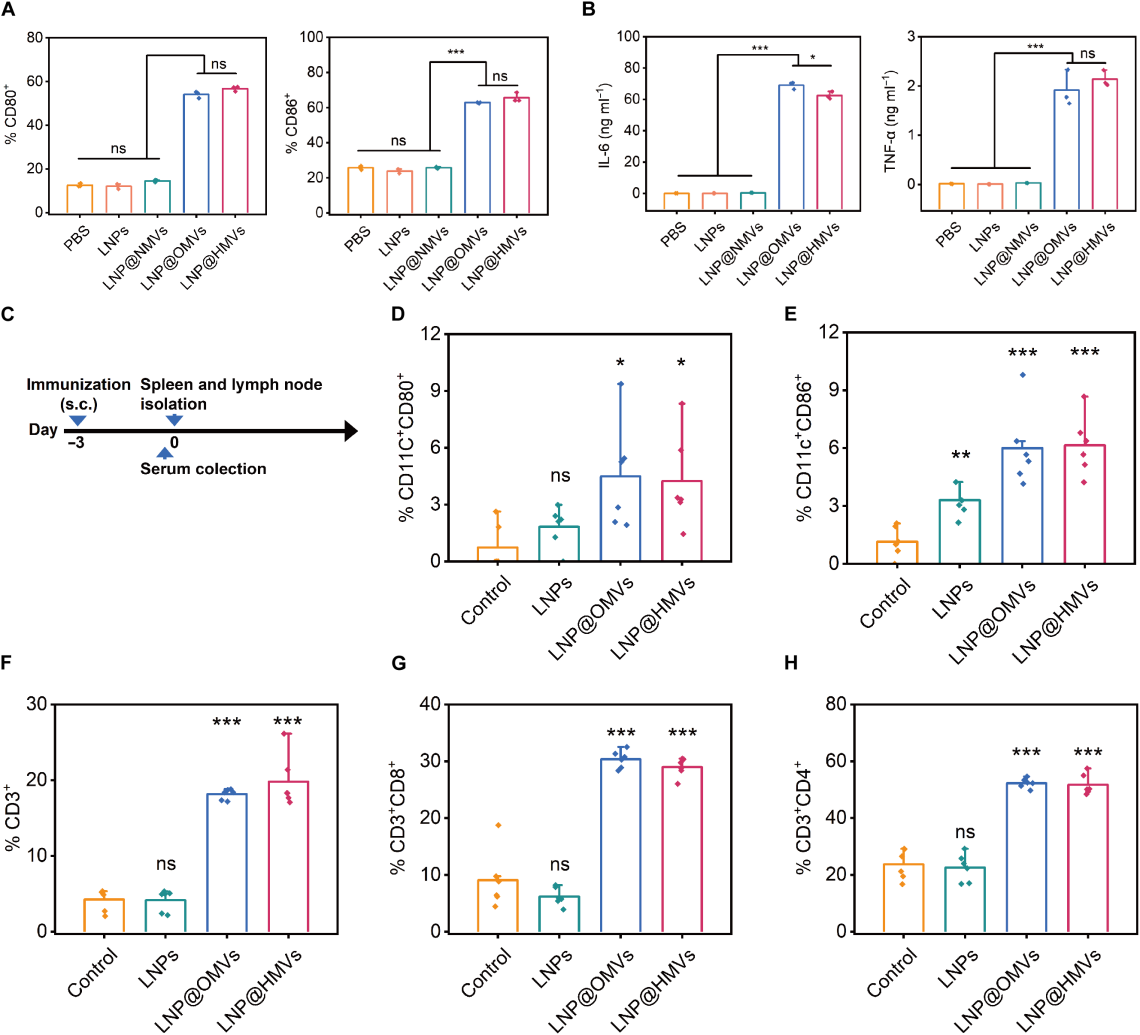
In vitro and in vivo immune responses activated by LNP@HMVs. (**A**) Expression levels of CD80^+^ and CD86^+^ in BMDCs (CD11c^+^) and (**B**) production of IL-6 and TNF-α of the cell supernatant measured by ELISA after different treatments (concentration, 2 μg ml^−1^ NPs) for 24 hours (*n* = 3). (**C**) Schematics of experimental design to evaluate the in vivo short-term immune responses activated by a single dose of various LNPs (20 μg/20 g) via subcutaneous injection. (**D**) Percentage of CD80^+^ and (**E**) CD86^+^ cells (gated on CD11c^+^ cells) in the LNs at day 3 after immunization (*n* = 6). (**F**) Percentage of CD3^+^ T cells, (**G**) CD8^+^ T cells (gated on CD3^+^ T cells), and (**H**) CD4^+^ T cells (gated on CD3^+^ T cells) in the spleen at day 3 after immunization (*n* = 6). Naive mice without immunization were used as control. *P < 0.05, **P < 0.01, and ***P < 0.001.

OMVs can be recognized by dendritic cells (DCs) and then trigger immune responses for bacteria elimination, and thus, they have been studied and even clinically used as vaccine to prevent bacterial infections (*17*). LNP@HMVs inherited the proteins and other biomacromolecules from OMVs on the surface (Fig. 2F). Consequently, they may retain the function of DC activation, which would be favorable for prophylactic protection against *E. coli* infection. To validate this assumption, the maturation of DCs in vitro was first characterized by analyzing the expression of costimulatory molecules CD80 and CD86 on bone marrow–derived DCs (BMDCs) (Fig. 6A and figs. S10 to S12). BMDCs treated with LNP@OMVs or LNP@HMVs showed elevated expressions of CD80 and CD86, indicating that they could activate the maturation of BMDCs. On the contrary, LNPs and LNP@NMVs could hardly facilitate the maturation of BMDCs with low expression of CD80 and CD86, similar to the PBS treatment group, indicating that LNPs and LNP@NMVs were not associated with immune activation. Consistent with the expression of costimulatory molecules, the secretion of immune cytokines including interleukin-6 (IL-6) and TNF-α from BMDCs after treatment with LNP@OMVs or LNP@HMVs was also higher than treatment with PBS, LNPs, and LNP@NMVs (Fig. 6B), further proving the activation of BMDCs upon treatment with LNP@OMVs or LNP@HMVs. The level of both costimulatory molecule expression and immune cytokine of BMDC displayed no significant difference between groups treated with LNP@OMVs and LNP@HMVs, which implied that the capability of OMVs on in vitro immune activation was not compromised upon hybridization with NMVs.

The in vivo immune responses upon subcutaneous injection of blank or membrane-functionalized LNPs (20 μg NPs/20 g) were then assessed by examining the activation of DCs in lymph nodes (LNs) and the percentage of T cell subsets in the spleen (Fig. 6C and fig. S10). Similar to in vitro data shown in Fig. 6A, LNP@HMVs induced higher frequencies of CD80^+^ and CD86^+^ DCs (CD11c^+^ cells) in LNs than the untreated control and LNP treatment groups (Fig. 6, D and E, and fig. S13), suggesting that LNP@HMVs increased the population of mature DCs in LNs, which is an essential step for inducing T cell and B cell responses. Subsequently, we detected the T cell (CD3^+^ cell) frequency in spleen and the percentage of T cell subsets (CD4^+^ and CD8^+^ in CD3^+^ cells) (Fig. 6, F to H, and fig. S14), as CD8^+^ T cells recognize and destroy infected cells by releasing various cytotoxins and CD4^+^ cells produce cytokines to assist cytotoxic T lymphocyte activation or support the development of humoral immunity driven by B cells to fight against bacterial infections. The percentage of CD3^+^ cells increased from 4.3% to 18.1 and 19.8% after immunization with LNP@OMVs and LNP@HMVs, respectively (Fig. 6F). The enhanced CD3^+^ cell level indicated that LNP@HMVs significantly increased the level of T cells in spleen. Moreover, the ratio of CD4^+^ in CD3^+^ cells increased from 23.7 to 51.8% (Fig. 6H), and CD8^+^ in CD3^+^ cells increased from 9.1 to 29.0% (Fig. 6G). The increasing ratio of CD4^+^ and CD8^+^ cells implied that LNP@HMVs activated specific cellular immunity in mice. Therefore, a single dose of LNP@HMVs, similar to LNP@OMVs, was capable of activating cellular immunity.

With short-term immunity of LNP@HMVs verified, we proceeded to investigate the long-term immune responses by subcutaneous vaccination of LNP@HMVs (20 μg/20 g) on day −28 and a booster vaccination on day −14 (fig. S15A). Two weeks after the booster vaccination, *E. coli*–specific antibody immunoglobulin G (IgG) titers induced by LNP@HMVs were determined to be significantly higher than the untreated group with approximately 20-fold increase (fig. S15B). The elevation of *E. coli*–specific antibody titers in the LNP@HMV-treated group indicated that LNP@HMVs induced long-term bacterium-specific B cell responses.

### In vivo prophylactic effects of LNP@HMVs

**Fig. 7.**
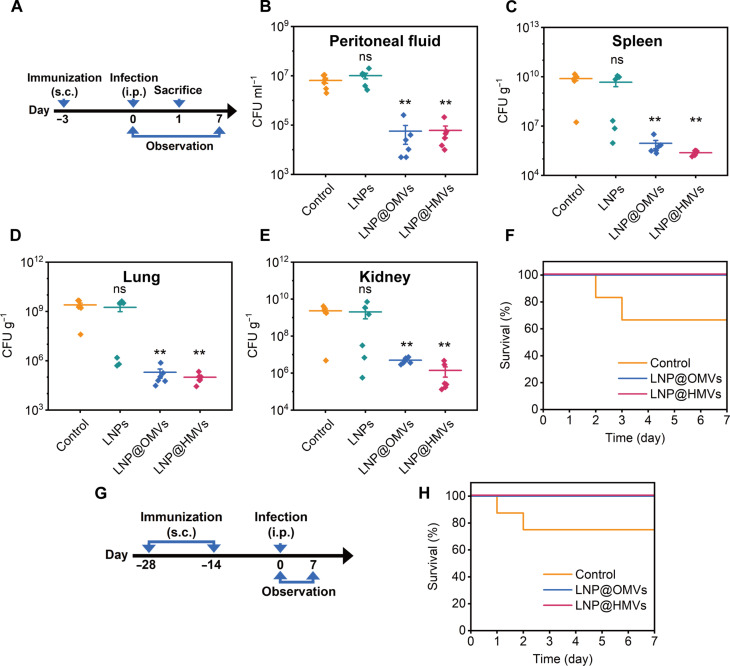
In vivo prophylactic effects of LNP@HMVs against *E. coli* infections. (**A**) Schematics of the short-term prophylactic experiment design of a single dose of LNP@OMVs or LNP@HMVs (20 μg/20 g). Quantification of the number of bacteria in (**B**) peritoneal fluid, (**C**) spleen, (**D**) lung, and **E**) kidney of the infected mice after different treatments. Infected mice without any treatment were used as control (*n* = 6). (**F**) Survival curves of the mice intraperitoneally infected with *E. coli* (1 × 10^7^ CFU/20 g) after different treatments (*n* = 6). (**G**) Schematics of the long-term prophylactic experiment design of two doses of LNP@OMVs or LNP@HMVs (20 μg/20 g). (**H**) Survival curves of the infected mice after different treatments (*n* = 8). Infected mice without any treatment were used as control. **P < 0.01.

After demonstrating that LNP@HMVs activated specific humoral and cellular immunity in vivo, we further evaluated their in vivo prophylactic effects against *E. coli* infection. First, the mice immunized subcutaneously with various LNPs (20 μg/20 g of mouse body weight) were infected by *E. coli* (1 × 10^7^ CFU/20 g, 100 μl) via intraperitoneal injection at day 3 after immunization (Fig. 7A). Comparing the number of bacteria present in the various tissues and organs of the infected mice, there was almost no bacterial reduction in the LNP group, while the counts of bacteria in the peritoneal fluid, spleen, lung, and kidney of the LNP@OMV and LNP@HMV groups were significantly reduced with approximately 2-log to 4-log reduction of bacterial CFU, suggesting that LNP@OMVs and LNP@HMVs enhanced the in vivo antibacterial potency of immune system (Fig. 7, B to E). The survivals of mice were recorded every 24 hours over a period of 7 days (Fig. 7F), and LNP@OMVs and LNP@HMVs showed excellent protective effect, maintaining 100% survival rate of the infected mice. Meanwhile, the control group with severe systemic *E. coli* infection exhibited 33.3% mortality rate within 3 days of infection. The improved survival rate was due to the effective in vivo immune activation (Fig. 7, D to H) that resulted in bacterial clearance in various tissue and organs of infected mice. Furthermore, the long-term prophylactic effect of LNP@HMVs against *E. coli* intraperitoneal infections was evaluated after subcutaneous immunization of various LNPs (20 μg/20 g) on day −28 and a booster vaccination on day −14 (Fig. 7G). Similar to the short-term prophylactic study, the immunization with LNP@OMVs or LNP@HMVs saved all the infected mice as compared to only 75% survival in the control group without any immunization (Fig. 7H), indicating that LNP@HMVs provided long-term prophylactic effect. Both in vitro and in vivo results proved that the LNP@HMVs inherited the immune activation effect of *E. coli*–derived OMVs and prevented the occurrence of *E. coli* infection.

## DISCUSSION

A neutrophil-bacterial LNP@HMV was successfully made for the dual-targeted antibiotic delivery and to activate specific humoral and cellular immunity for prophylactic prevention of Gram-negative bacterial infections. LNP@HMVs obtained from the coating of LNPs with the hybrid membrane of bacterial OMVs and NMVs targeted inflammatory vascular cells and Gram-negative bacteria, resulting in enhanced accumulation at the infection site. With the targeting properties to both infection microenvironment and bacteria, LNP@HMVs loaded with the antibiotic Nor exhibited enhanced uptake efficiency of antibiotic by bacteria. Compared with Nor-encapsulated LNPs with mono-membrane coating (OMVs or NMVs), LNP@HMVs showed enhanced elimination of planktonic bacteria and biofilms in vitro and a stronger therapeutic effect in treating mice with systemic or lung infection in vivo even at low antibiotic doses. Moreover, LNP@HMVs acted as an antibacterial vaccine by activating specific humoral and cellular immunity, preventing the occurrence of bacterial infections. Overall, this therapeutic nanoparticulate system has great potential to specifically transport antibiotics to inflammatory tissues and bacteria for the effective treatment of bacterial infections, and to activate the immunity as vaccine to prevent bacterial infections.

## MATERIALS AND METHODS

### Materials

GMS and Nor were obtained from Aladdin, Shanghai, China. Poloxamer 188 was obtained from Beijing Probe Bioscience Co. Ltd., Beijing, China. Bicinchoninic acid assay kit, SDS–polyacrylamide gel electrophoresis (PAGE) kit, 3-(4,5-dimethylthiazol-2-yl)-2,5-diphenyltetrazolium bromide (MTT) kit, DiI, and DiO were obtained from Beyotime Biotechnology, Shanghai, China. Mueller-Hinton broth (MHB) and agar were obtained from BD Difco, USA. RPMI 1640 cell culture medium was obtained from Gibco, Thermo Fisher Scientific, USA. IL-6 and TNF-α enzyme-linked immunosorbent assay (ELISA) kits were obtained from NeoBioscience Co. Ltd., Shenzhen, China. LIVE/DEAD BacLight bacterial viability kit (L-7012) was bought from Invitrogen, Thermo Fisher Scientific, USA. Fluorescein isothiocyanate (FITC)–conjugated anti-mouse CD11c, phycoerythrin (PE)–conjugated anti-mouse CD80, PE-conjugated anti-mouse CD86, allophycocyanin (APC)–conjugated anti-mouse CD3, PE-conjugated anti-mouse CD8a, and FITC-conjugated anti-mouse CD4 were purchased from Tonbo Biosciences, USA. Murine IL-4, murine granulocyte-macrophage colony-stimulating factor (GM-CSF), and human TNF-α were bought from PeproTech, USA. *E. coli* (ATCC no. 25922), *S. aureus* (ATCC no. 6538), *K. pneumoniae* (ATCC no. 4352), and HL-60 (ATCC no. CCL-240) were obtained from the American Type Culture Collection (ATCC).

### Isolation of *E. coli*–derived OMVs and NMVs

*E. coli* (strain ATCC25922) were cultured in MHB medium under shaking at 37°C overnight. The suspension was centrifuged at 6000*g* for 10 min to remove *E. coli* cells. Subsequently, the supernatant was ultracentrifuged at 100,000*g* for 1 hour at 4°C. The obtained OMVs were washed with PBS thrice and resuspended in PBS.

HL-60 cells were differentiated to neutrophil-like cells by incubation in RPMI 1640 medium containing dimethyl sulfoxide at 1.3% (v/v) for 3 days and activated by lipopolysaccharide (1 μg ml^−1^) for 4 hours. Cells were suspended in a lysing buffer that contains tris-HCl (pH 7.5, 30 mM), d-mannitol (225 mM), sucrose (75 mM), EDTA (0.2 mM), and a protease inhibitor cocktail. Subsequently, cells were homogenized through a Dounce homogenizer with a pestle for 30 passes. The homogenized suspension was centrifuged at 3000*g* for 10 min to remove the pellet. Then, NMVs were collected from the supernatant centrifuged at 20,000*g* for 1 hour and washed twice with PBS. Last, NMVs were suspended in PBS.

### Preparation and characterization of HMVs of OMVs and NMVs

OMVs (100-μg protein weight) and NMVs (100-μg protein weight) were mixed together and sonicated for 10 min. The solution was then extruded through 800- and 400-nm polycarbonate membrane using an Avanti mini-extruder to form HMVs. A drop of samples was precipitated on a carbon-coated grid and stained with 1% phosphotungstic acid for observation under a transmission electron microscope (TEM; FEI Tecnai G2 Spirit, FEI, USA).

DiO (λ_excitation/emission_ = 484/501 nm) and DiI (λ_excitation/emission_ = 550/567 nm) were used to conduct the FRET study. NMVs were added to the DiO/DiI-doped OMVs at NMV to OMV protein weight ratios of 0:1, 2:1, 4:1, respectively. Then, the mixture was sonicated for 10 min to facilitate membrane fusion. The fluorescence spectrum of each sample was read between 500 and 650 nm using a fluorescence spectrophotometer (FL-970, Techcomp, China) with an excitation wavelength of 490 nm.

Moreover, the membrane fusion was also confirmed through the observation under a CLSM (Zeiss, LSM880, Germany). OMVs were stained with DiO (Green), and NMVs were stained with DiI (Red). The HMVs were obtained through sonication and subsequent extrusion of the mixture of OMVs and NMVs. Then, the HMVs or simple mixtures without sonication and extrusion were imaged by CLSM.

### Preparation and characterization of LNP-N@HMVs

A solvent diffusion method was applied to prepare the LNP-Ns as reported previously (*33*). Briefly, GMS (15 mg) and Nor (2.1 mg) were mixed and dissolved in ethanol (1.5 ml) at 85°C. Poloxamer188 (15 mg) was dissolved in double-distilled water (ddH_2_O, 15 ml) at 75°C. Subsequently, the aqueous phase and organic phase were mixed at 75°C under the condition of 400 rpm stirring. After stirring for 10 min, the prepared solution was cooled down at ice bath to solidify the melted lipid droplets.

To prepare membrane-coated LNP-Ns, OMVs (100-μg protein weight) and NMVs (100-μg protein weight) were mixed together and sonicated for 10 min. The solution was then mixed with 200-μg weight of LNP-Ns and extruded through 800- and 400-nm polycarbonate membrane using an Avanti mini-extruder to form hybrid membrane–coated nanoparticle LNP-N@HMVs. The nanoparticles coated with OMVs or NMVs were fabricated through the same method.

A drop of samples was precipitated on a carbon-coated grid and stained with 1% phosphotungstic acid for TEM observation. Hydrodynamic size and surface zeta potential were measured by dynamic light scattering on a Zetasizer Nano ZS (Malvern). High-performance liquid chromatography (LC-20AT, Shimadzu, Japan) was used to test the content of Nor according to the protocol described in the *Pharmacopoeia of the People’s Republic of China* (2020 edition).

### Uptake of nanoparticles by inflammatory HUVECs

HUVECs were cultured in Dulbecco’s modified Eagle’s medium (DMEM) containing recombinant human TNF-α (50 ng ml^−1^) at 37°C. After 4 hours of stimulation, the cells were washed with PBS and then incubated with dye-labeled nanoparticles (20 μg ml^−1^) in DMEM for 0.5 or 1 hour. After incubation, the cells were washed three times with ice-cold PBS. The cells were stained with Hoechst for 10 min, washed three times with ice-cold PBS, and imaged under CLSM. For flow cytometric analysis, cells were trypsinized and washed with PBS, followed by dispersion in PBS and analysis on a flow cytometer (CytoFLEX, Beckman, USA).

### Penetration of nanoparticles across the inflammatory HUVEC layers

HUVECs were seeded in the upper chamber of the transwell (3-μm pore polycarbonate membrane inserts, 10^6^ cells per chamber) and incubated with TNF-α (50 ng/ml) for 4 hours. Then, LNP-Ns or LNP-N@HMVs (rhodamine B–labeled LNPs) were added in the upper chamber. After incubation for 3 or 6 hours, the solution in the bottom chamber was taken to measure the fluorescence intensity of rhodamine by a fluorescence spectrophotometer (FL-970, Techcomp, China).

### Uptake of nanoparticles by bacteria

Bacteria *E. coli* (ATCC no. 25922), *S. aureus* (ATCC no. 6538), or *K. pneumoniae* (ATCC no. 4352) cultured in MHB medium overnight were diluted to ~10^8^ CFU ml^−1^ and washed with PBS, followed by incubation with dye-labeled nanoparticles (20 μg ml^−1^) in PBS at 37°C for 1 hour. Next, the bacteria were washed with PBS three times. The bacteria were stained with Hoechst for 10 min and washed three times before dispersion in PBS. Suspensions of samples (10 μl) were placed onto the glass slide and imaged under CLSM. For flow cytometric analysis, the bacteria were collected after PBS washing and then analyzed with a flow cytometer (CytoFLEX, Beckman, USA).

### In vitro antibacterial and antibiofilm assay

To investigate the antibacterial activity of LNP-N@HMVs, *E. coli* cells (~10^5^ CFU ml^−1^) were incubated with Nor, LNP-Ns, or LNP-N@HMVs at 37°C for 8 hours at an equivalent Nor concentration (0.12 μg ml^−1^). *K. pneumoniae* cells (~10^5^ CFU ml^−1^) were incubated with Nor, LNP-Ns, or LNP-N@HMVs at 37°C for 8 hours at an equivalent Nor concentration (1 μg ml^−1^). The obtained suspension was serially diluted and streaked on MHB agar plates. Bacterial CFU counting and photographing were carried out after incubation at 37°C for 18 to 24 hours.

The Live/Dead BacLight bacterial viability kit was further used to evaluate antibacterial efficacy. The kit contains propidium iodide (PI) and SYTO9 fluorescent dye, where red dye PI stains bacteria with disrupted cell membrane and green dye SYTO9 stains all the bacteria. *E. coli* cultured overnight were washed twice with PBS and resuspended in fresh MHB medium. Then, the *E. coli* suspension (~10^8^ CFU ml^−1^, 500 μl) was mixed with Nor, LNP-Ns, or LNP-N@HMVs at an equivalent Nor concentration (0.24 μg ml^−1^, 500 μl) and incubated at 37°C for 6 hours. After incubation, the bacterial suspension was washed with PBS three times and stained with PI and SYTO9 according to the BacLight bacterial viability kit manual. The viability of *E. coli* was observed under CLSM.

To test the antibiofilm activity of LNP-N@HMVs, *E. coli* (~10^8^ CFU ml^−1^) were cultured in 96-well plates at 37°C for 48 hours to form biofilms. Subsequently, the medium was removed and different samples at equivalent Nor concentration (0.2, 0.5, 1.0, and 2.0 μg ml^−1^) were added into each well and incubated for 24 hours. After incubation, the medium was removed and the biofilms were stained with crystal violet (0.1%) for 30 min and washed with ddH_2_O. Last, the crystal violet was dissolved in ethanol and the biomass of the biofilm was quantified by recording and calculating according to the absorbance at 570 nm.

### In vitro and in vivo biocompatibility of LNP-N@HMVs

The cytotoxicity of LNPs and LNP@HMVs toward HUVECs was evaluated through MTT method. HUVECs (10^5^ cells ml^−1^) were seeded into 96-well plates and cultured for 24 hours. LNPs and LNP@HMVs at different concentrations of LNPs (concentration ranging from 10 to 160 μg ml^−1^) were added to each well and incubated for 24 hours. HUVECs without any treatment were used as a control. After incubation, MTT solution (100 μl, 0.5 mg ml^−1^) was added to each well and incubated in the dark for 4 hours. Formazan solution (100 μl) was further added to dissolve formazan for 4 hours. Last, the viability of HUVECs was measured by recording and calculating according to the absorbance at 570 nm through a microplate reader (Thermo Fisher Scientific, Multiskan FC, USA).

All the in vivo experiments performed were approved by the Institutional Animal Care and Use Committee of School of Pharmaceutical Sciences (Shenzhen), Sun Yat-sen University (approval no. SYSU-YXYSZ-20220308) and Guangzhou Institutes of Biomedicine and Health, Chinese Academy of Science (approval nos. N2022137 and N2022138). To evaluate the in vivo biocompatibility, C57BL/6 mice (6 to 8 weeks) were randomly divided into two groups (*n* = 4) and treated with LNP-N@HMVs (25 μg/20 g) or PBS via tail vein injection, respectively. After 7 days, the sera were collected for biochemical analysis including ALT, AST, UREA, and CREA to evaluate the functions of liver and kidney.

### In vivo imaging of LNP@HMVs in mouse bacterial infection models

A muscle infection model was used to evaluate the in vivo targeting ability of the LNP@HMVs upon tail vein injection. The mice (*n* = 3) were shaved and then injected intramuscularly with *E. coli* (1 × 10^8^ CFU/20 g, 50 μl) on the right thigh, namely, the infected site. After 2 hours of infection, the mice were injected with LNPs, LNP@NMVs, LNP@OMVs, or LNP@HMVs via tail vein injection and imaged at predetermined time points (3, 6, 9, and 24 hours) using a small animal living imaging system IVIS (IVIS Lumina III, PerkinElmer, USA; λex/λem = 750/790 nm).

A lung infection model was also used to evaluate the in vivo targeting ability of the LNP@HMVs upon intratracheally administration. The mice (*n* = 3) were intratracheally inoculated with *K. pneumoniae* (1 × 10^5^ CFU/20 g, 50 μl). After 2 hours of infection, the mice were intratracheally administered with LNPs or LNP@HMVs and euthanized at predetermined time points (3, 6, 9, and 24 hours). The lungs were collected for analysis using the IVIS (IVIS Lumina III, PerkinElmer, USA; λex/λem = 750/790 nm).

### In vivo therapeutic effect of bacterial infection

To evaluate the in vivo therapeutic efficacy of LNP@HMVs, C57BL/6 mice (6 to 8 weeks) were treated with *E. coli* (1 × 10^7^ CFU/20 g, 100 μl) intraperitoneally to establish an acute peritonitis infection model. The mice (*n* = 5) were administrated intravenously with Nor, LNPs, LNP@NMVs, LNP@OMVs, or LNP@HMVs (25 μg Nor/20 g) after infection with *E. coli* intraperitoneally for 2 hours. After 24 hours of infection, the mice were euthanized and intraperitoneally injected with PBS (5 ml) for the collection of peritoneal fluid. Subsequently, the spleen, lung, and kidney were collected and homogenized for 1 min. The peritoneal fluid and homogenized suspension of different organs were serially diluted and streaked on MHB agar plates for CFU counting.

C57BL/6 mice (6 to 8 weeks) were treated with *K. pneumoniae* (1 × 10^5^ CFU/20 g, 50 μl) intratracheally to establish a lung infection model. The mice (*n* = 5) were administrated intratracheally with Nor, LNPs, or LNP@HMVs (1 μg Nor/20 g) after infection with *K. pneumoniae* intratracheally for 2 hours. At 8 hours after infection, the mice were euthanized and the lungs were collected and homogenized for CFU counting.

### In vitro BMDC maturation assays

BMDCs were collected from bone marrow cells of male C57BL/6 mice (6 to 8 weeks) and differentially induced by GM-CSF and IL-4. In brief, the cells in femurs and tibias of mice were collected by gently flushing with PBS and washed twice by centrifugation at 2000 rpm for 5 min. The collected cells were then cultured in RPMI 1640 medium containing 10% fetal bovine serum and murine GM-CSF (20 ng ml^−1^) and murine IL-4 (10 ng ml^−1^). After culture for 6 days, the BMDCs were collected and incubated with different samples at a final protein concentration of 2 μg ml^−1^ at 37°C for 24 hours. The BMDCs were then collected and labeled by FITC-conjugated anti-mouse CD11c, PE-conjugated anti-mouse CD80, or PE-conjugated anti-mouse CD86. Last, the maturation of BMDCs was analyzed using a flow cytometer (CytoFLEX, Beckman, USA). The gating strategy was shown in fig. S10. In addition, the supernatant of BMDCs after incubation was collected to evaluate the levels of IL-6 and TNF-α by ELISA kits.

### In vivo immunization assays and prophylactic effect against bacterial infection

C57BL/6 mice (6 to 8 weeks) were immunized via subcutaneous injection of various LNPs (20 μg/20 g). At day 3 after injection, serum, LNs, and spleen were collected for the immunization assays including evaluation of IgG titers in the serum, DC maturation in the LNs, and the percentage of T cell subsets in the spleen.

To evaluate the IgG titers, a 96-well plate was coated with 100-ng lysate of *E. coli* overnight and washed with PBS thrice. Next, the plate was blocked with 2 wt % bovine serum albumin for 1 hour and washed with PBS thrice. Serially diluted serum samples were added to each well for 1-hour incubation and washed with PBS for three times, followed by the addition of horseradish peroxidase–conjugated goat anti-mouse IgG (1:5000). After washing by PBS, the plate was reacted with trimethylboron substrate for 10 min and terminated by 1 M HCl. Last, IgG titers were quantified by observing the absorbance at 450 nm.

To measure the maturation of DCs, the collected LNs were ground by using syringe piston at ice bath. Individual cell suspensions were obtained by passing through a 70-μm cell strainer and washed once with PBS. The collected cells were then stained with FITC-conjugated anti-mouse CD11c, PE-conjugated anti-mouse CD80, or PE-conjugated anti-mouse CD86 at 4°C for 30 min. The data were acquired on a flow cytometer (CytoFLEX, Beckman, USA). The gating strategy was shown in fig. S10.

To test the frequency of T cells and their subsets, the collected spleens were ground and passed through a 70-μm cell strainer to obtain individual spleen cells. The cell suspensions were stained with APC-conjugated anti-mouse CD3, PE-conjugated anti-mouse CD8a, and FITC-conjugated anti-mouse CD4 for 30 min at 4°C. The data were acquired on a flow cytometer (CytoFLEX, Beckman, USA). The gating strategy was shown in fig. S10.

The in vivo prophylactic ability of LNP@HMVs was verified on the C57BL/6 mice (6 to 8 weeks) immunized via subcutaneous injection of various LNPs (20 μg/20 g). At day 3 after immunization, the mice were infected by *E. coli* (1 × 10^7^ CFU/20 g, 100 μl) intraperitoneally. After 24 hours of infection, the mice were euthanized and intraperitoneally injected with PBS (5 ml) to collect the peritoneal fluid. Subsequently, the spleen, lung, and kidney were collected and homogenized in ice-cold PBS. The peritoneal fluid and homogenized suspensions were serially diluted and plated for CFU counting.

To evaluate the long-term in vivo prophylactic efficacy of LNP@HMVs, C57BL/6 mice (6 to 8 weeks) were immunized via subcutaneous injection of various LNPs (20 μg/20 g) on day −28 and a booster vaccination on day −14. Two weeks after the booster vaccination, the mice were infected by *E. coli* (1 × 10^7^ CFU/20 g, 100 μl) intraperitoneally. The survival rate was monitored daily.

### Statistical analysis

The significant difference was analyzed by Student’s *t* test. The experiments in this work were performed at least with triplicates, and the results were presented as means ± SD. Significance was denoted by ns (nonsignificance), *P < 0.05, **P < 0.01, ***P < 0.001. All data were analyzed by SPSS Software.
