# Supplementary material for: Biofunctional lipid nanoparticles for precision treatment and prophylaxis of bacterial infections
Source: Sci Adv. 2024 Apr 5;10(14):eadk9754. doi: 10.1126/sciadv.adk9754 (PMC10997193; doi:10.1126/sciadv.adk9754)
Supplement: Supplementary file 1 — Figs. S1 to S15 Tables S1 to S4 [file sciadv.adk9754_sm.pdf]

Science Advances
AAAS

Supplementary Materials for

Biofunctional lipid nanoparticles for precision treatment and prophylaxis of bacterial infections

Xinran Peng *et al.*

Corresponding author: Xin Ding, dingxin3@mail.sysu.edu.cn; Yi Yan Yang, yyyang@bti.a-star.edu.sg; Peiyan Yuan, yuanpy3@mail.sysu.edu.cn

Sci. Adv. **10**, eadk9754 (2024)
DOI: 10.1126/sciadv.adk9754

This PDF file includes:

Figs. S1 to S15
Tables S1 to S4

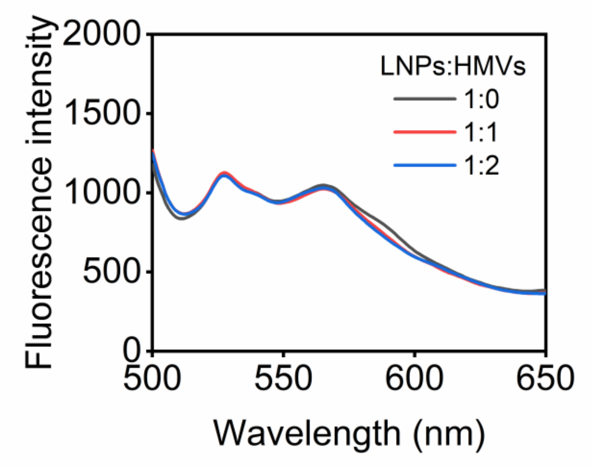

Fig. S1.

Fluorescent spectra of LNPs labelled with FRET dye pair (DiO and DiI) after sonication and extrusion with HMVs at different weight ratios.

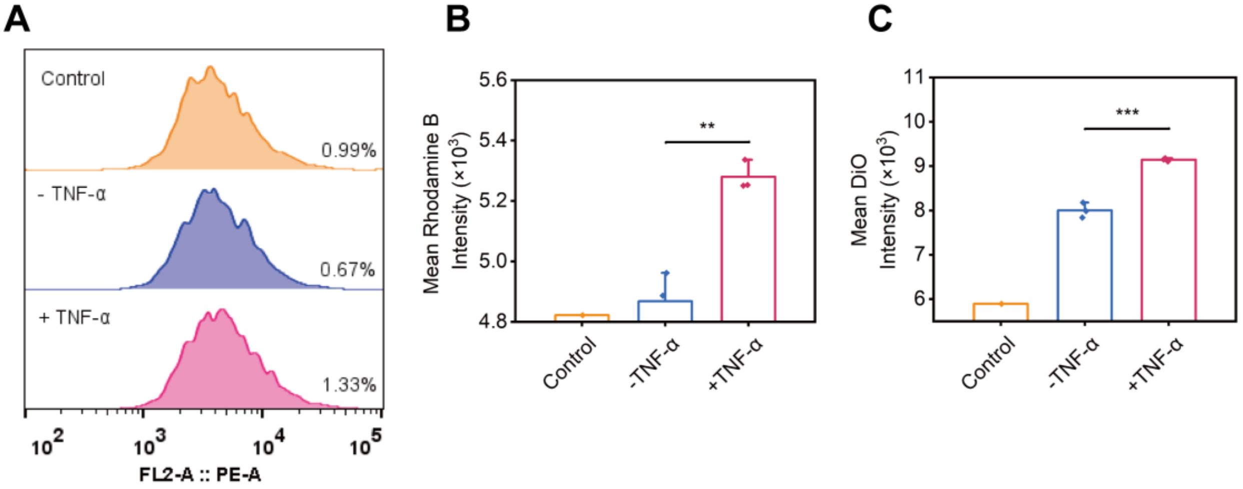

Fig. S2.

(**A**) Flow cytometry analysis of non-activated or TNF-α-activated HUVECs cultured with LNP-N@HMVs (Concentration: 20 μg mL^{-1} NPs) for 0.5 h, where Rhodamine B and DiO were used to stain LNPs and HMVs, respectively. The mean fluorescence intensity of Rhodamine B (**B**) and DiO (**C**) in non-activated or TNF-α-activated HUVECs. HUVECs without any treatment were set as control (n = 3, $^{**}p < 0.01$, and $^{***}p < 0.001$).

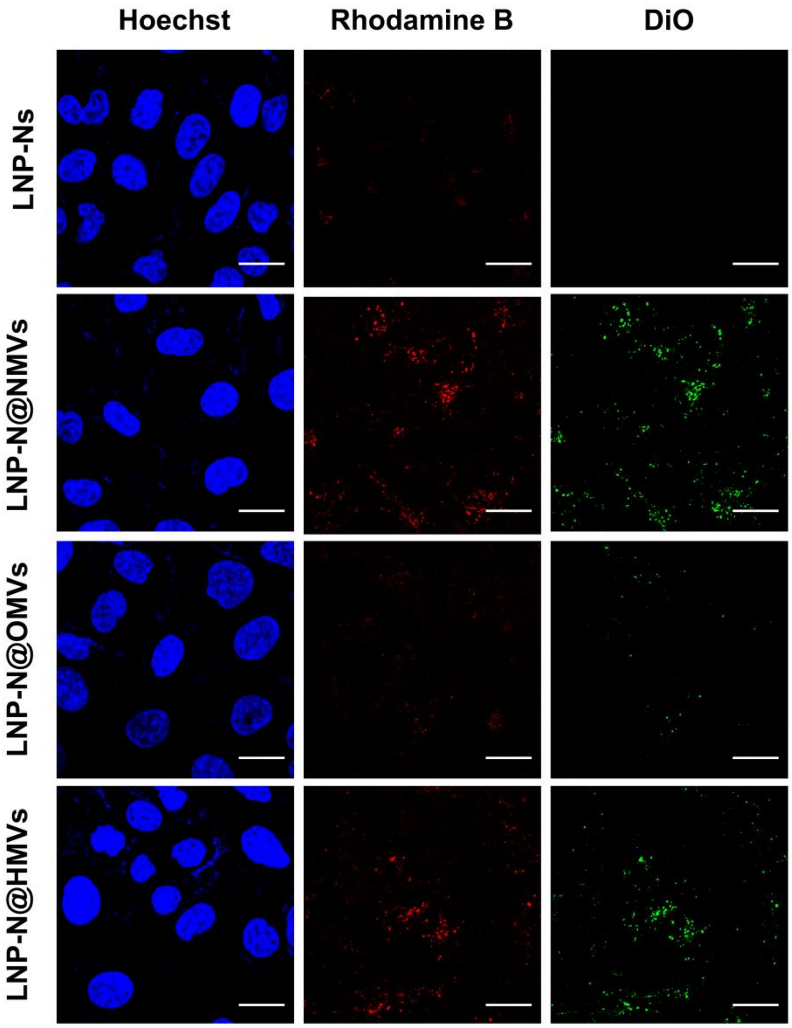

Fig. S3.

Confocal microscopic images of TNF-α-activated HUVECs cultured with LNP-Ns, LNP-N@NMVs, LNP-N@OMVs or LNP-N@HMVs (Concentration: 20 μg mL^{-1} NPs) for 1 h (Scale bars = 20 μm). The merge images were shown in main text **Fig. 2B**.

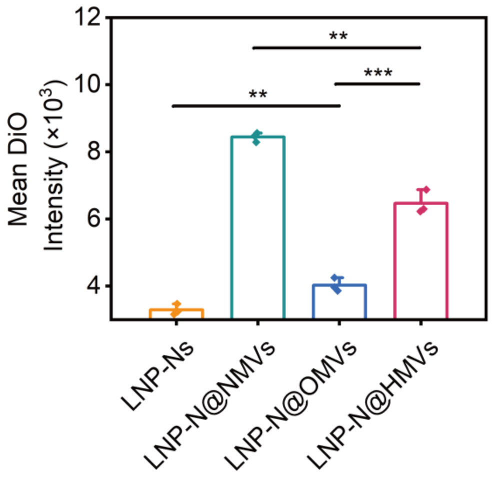

Fig. S4.

Uptake of different LNPs (Concentration: 20 μg mL^{-1} NPs) by HUVECs characterized by the mean fluorescence intensity of HMVs labelled with DiO, in TNF-α-activated HUVECs, and HUVECs without any treatment were used as control (n = 3, **$p < 0.01$, and ***$p < 0.001$).

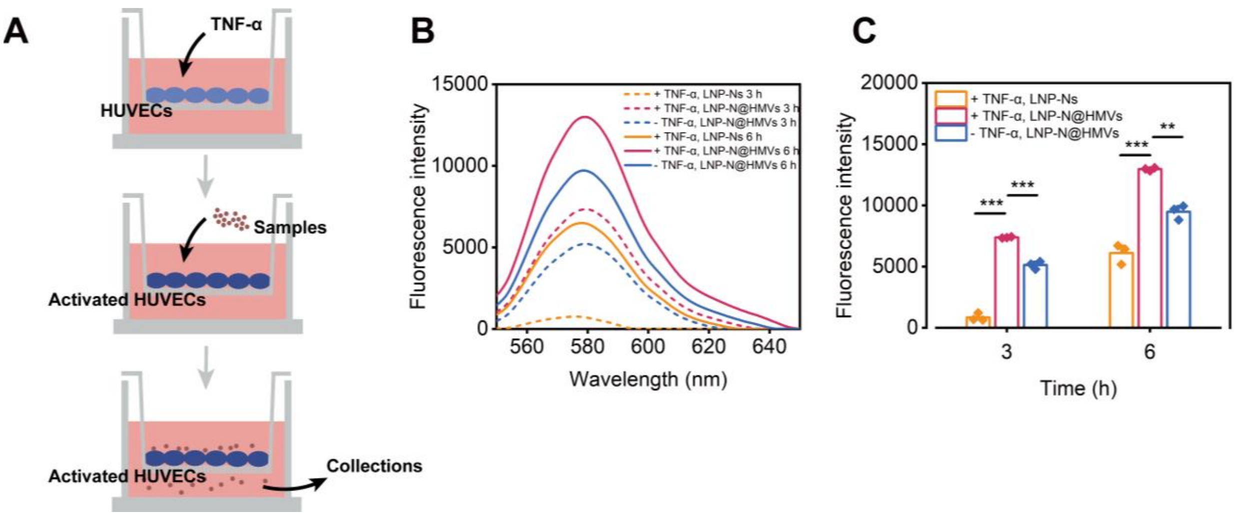

Fig. S5.

Capacity of LNP-N@HMVs to target and traverse the inflammatory vascular endothelial cells. (A) Schematic representation of transwell assay. (B) Fluorescent spectra and (C) fluorescent intensity at 580 nm of the suspension collected in the lower chamber of the transwell in different groups: TNF-α-activated HUVECs incubated with LNP-Ns, TNF-α-activated HUVECs incubated with LNP-N@HMVs, non-activated HUVECs incubated with LNP-N@HMVs for 3 h or 6 h. (n = 3, $**p < 0.01$, and $***p < 0.001$).

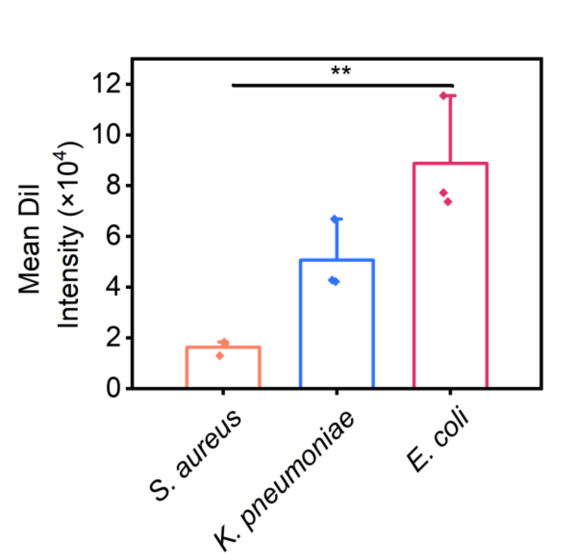

Fig. S6.

Uptake of *E. coli*-derived OMVs (Concentration: 20 μg mL^{-1}) by different bacteria. Mean fluorescence intensity of DiI which was used to label OMVs in *E. coli*, *K. pneumoniae* and *S. aureus* (n = 3, $^{**}p < 0.01$).

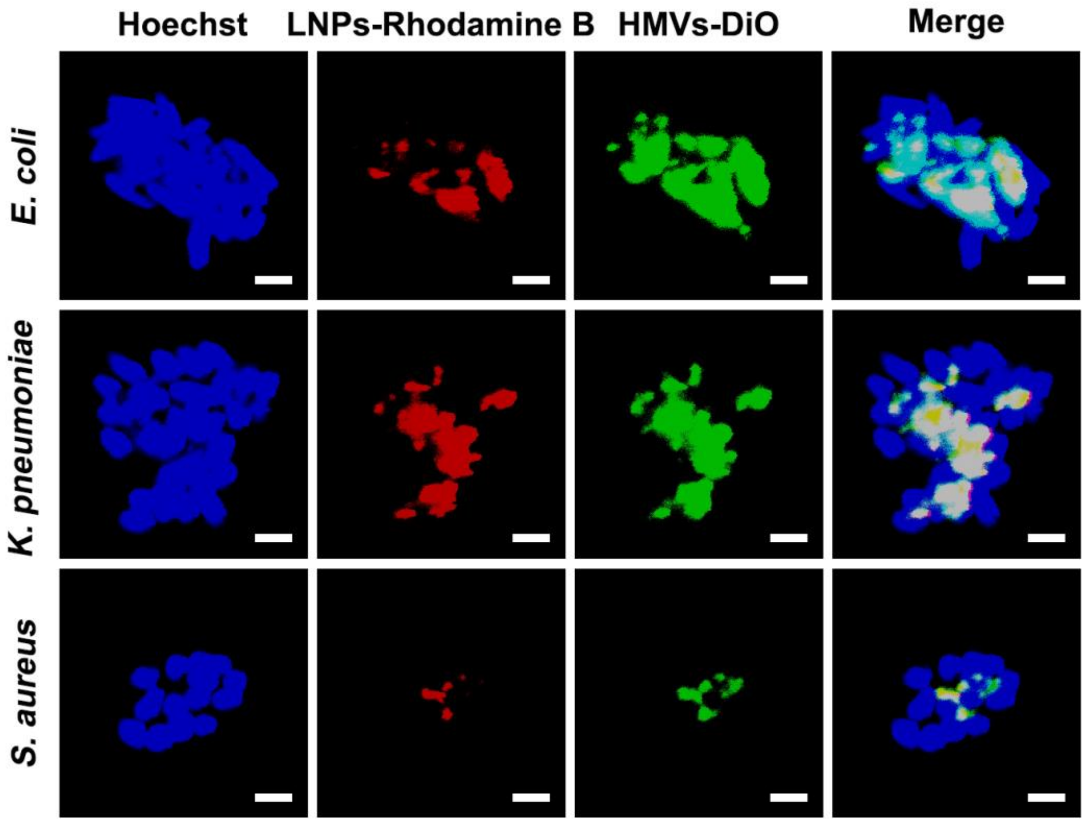

Fig. S7.

Confocal microscopic images of *E. coli*, *K. Pneumoniae* and *S. aureus* cultured with LNP@HMVs (Concentration: 20 μg mL^{-1} NPs) for 1 h (Scale bars = 2 μm).

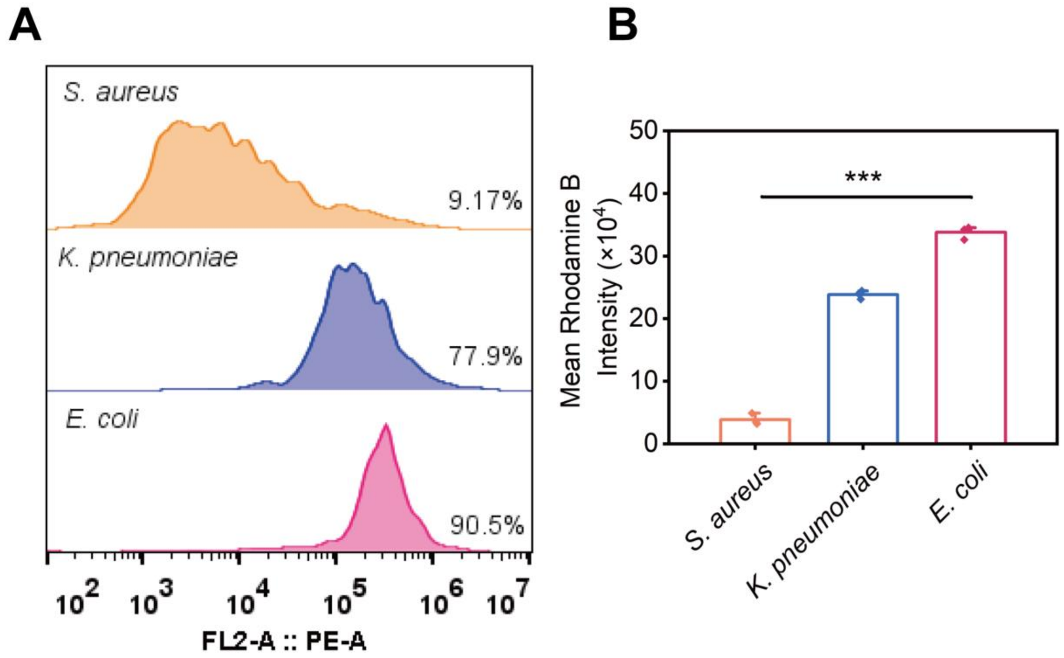

Fig. S8.

(**A**) Flow cytometry analysis of different bacteria cultured with LNP@HMVs (Concentration: 20 μg mL^{-1} NPs) for 1 h, where Rhodamine B was used to label LNPs. (**B**) The mean fluorescence intensity of Rhodamine B in LNP@HMVs-treated *E. coli*, *K. pneumoniae* and *S. aureus* (n = 3, ***p < 0.001).

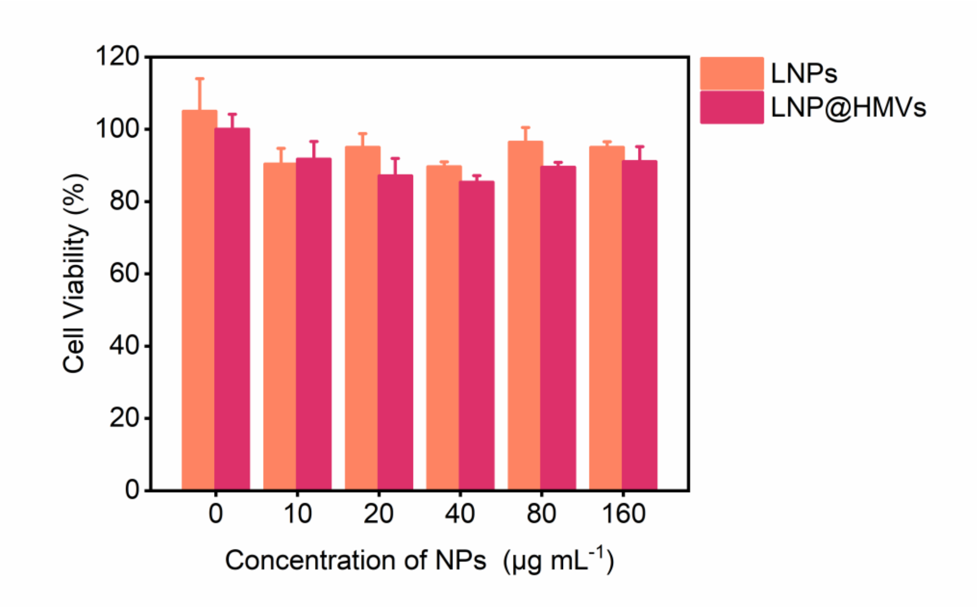

Fig. S9.

Viability of HUVECs cultured with different concentrations of LNPs or LNP @HMVs for 24 h at 37ºC (n = 3).

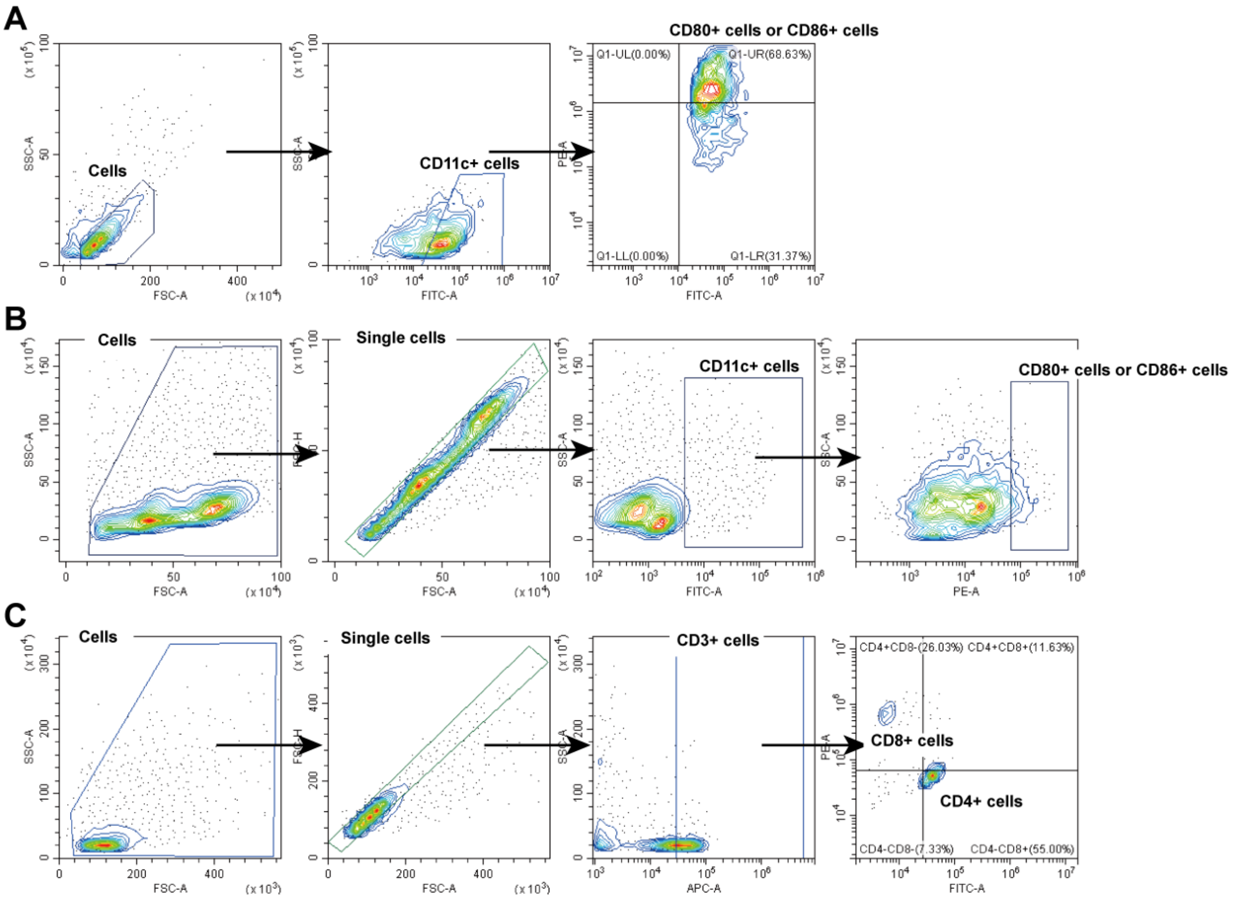

Fig. S10.

(A) Gating strategy of BMDCs after different treatments in vitro for 24 h (Fig. 6A). (B) Gating strategy of DCs after different treatments in vivo at day 3 post immunization (Fig. 6D and 6E). (C) Gating strategy of T cells after different treatments in vivo at day 3 post immunization (Fig. 6F to 6H).

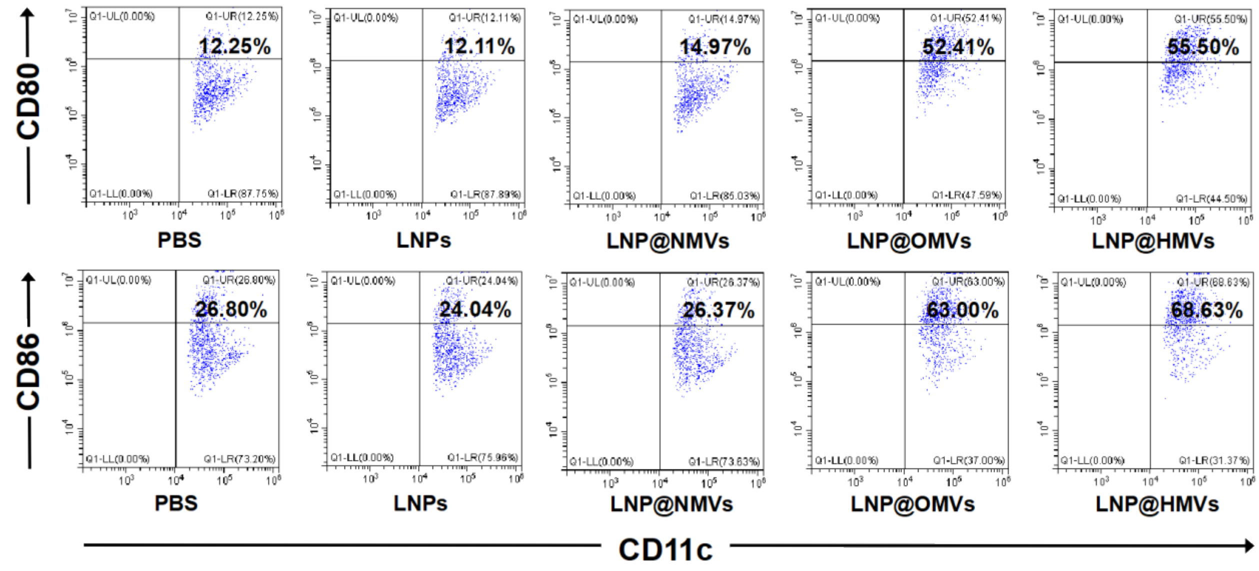

Fig. S11.

Expression levels of CD80+ and CD86+ in BMDCs (CD11c+) after different treatments *in vitro* for 24 h.

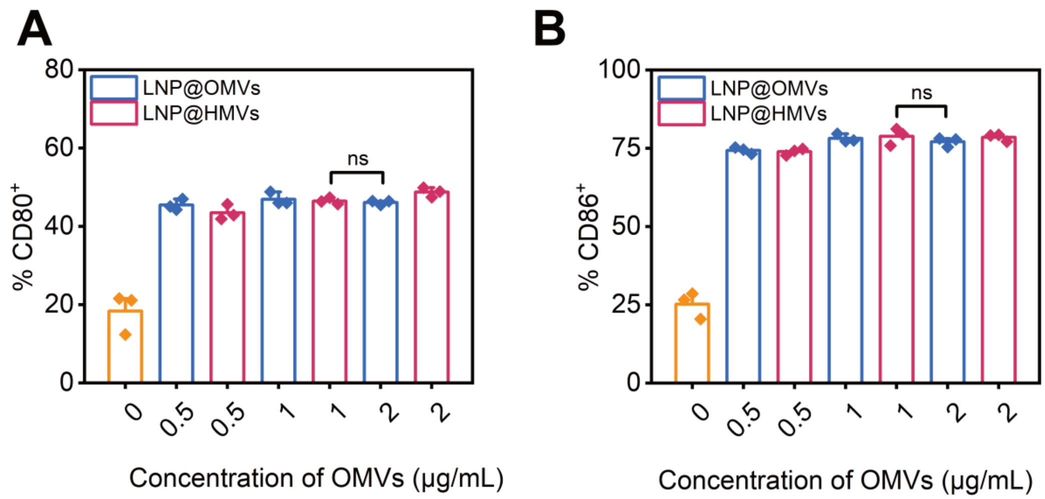

Fig. S12.

Expression levels of $CD80^+$ (A) and $CD86^+$ (B) in BMDCs ($CD11c^+$) after treatment with LNP@OMVs or LNP@HMVs (concentration: 0.5 - 2 μg mL^{-1} OMVs) for 24 h (n = 3, ns representing non-significance).

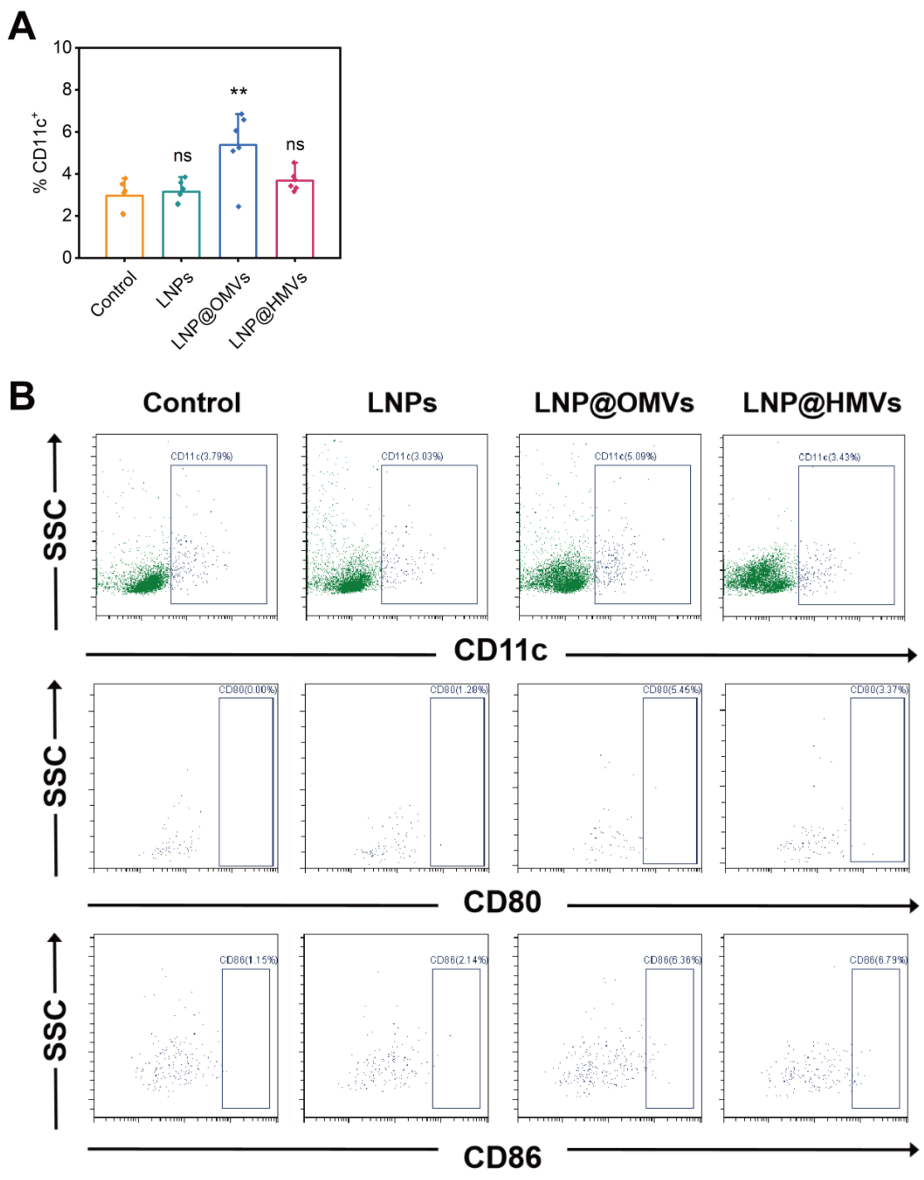

Fig. S13.

(**A**) Percentage of CD11c$^+$ cells in the lymph nodes characterized by flow cytometry at day 3 post immunization ($n = 6$, *$p < 0.05$, and ns representing non-significance).

(**B**) Percentage of CD11c$^+$ cells, percentage of CD80$^+$ cells (gated on CD11c$^+$ cells), and percentage of CD86$^+$ cells (gated on CD11c$^+$ cells) in the lymph nodes characterized by flow cytometry at day 3 post immunization.

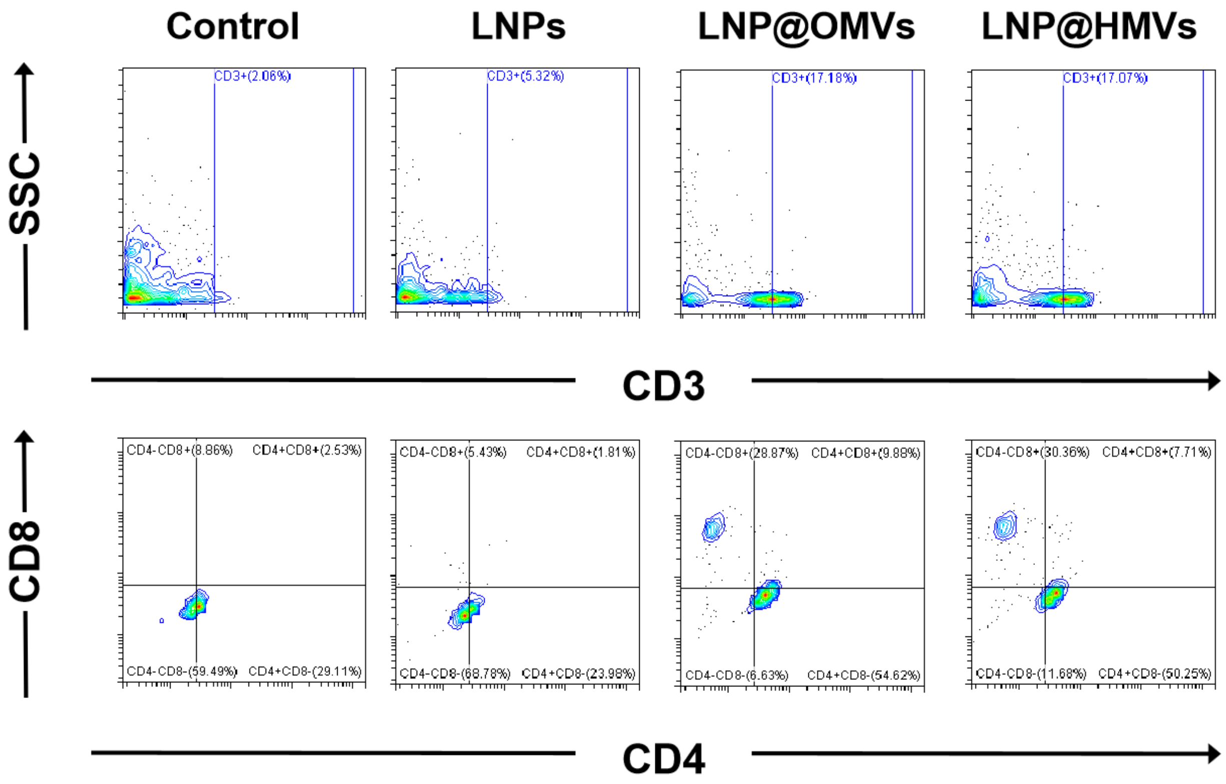

Fig. S14.

Percentage of $CD3^+$ T cells, percentage of $CD4^+CD8^-$ T cells (gated on $CD3^+$ T cells), and percentage of $CD4^-CD8^+$ T cells (gated on $CD3^+$ T cells) in the spleen characterized by flow cytometry at day 3 post immunization.

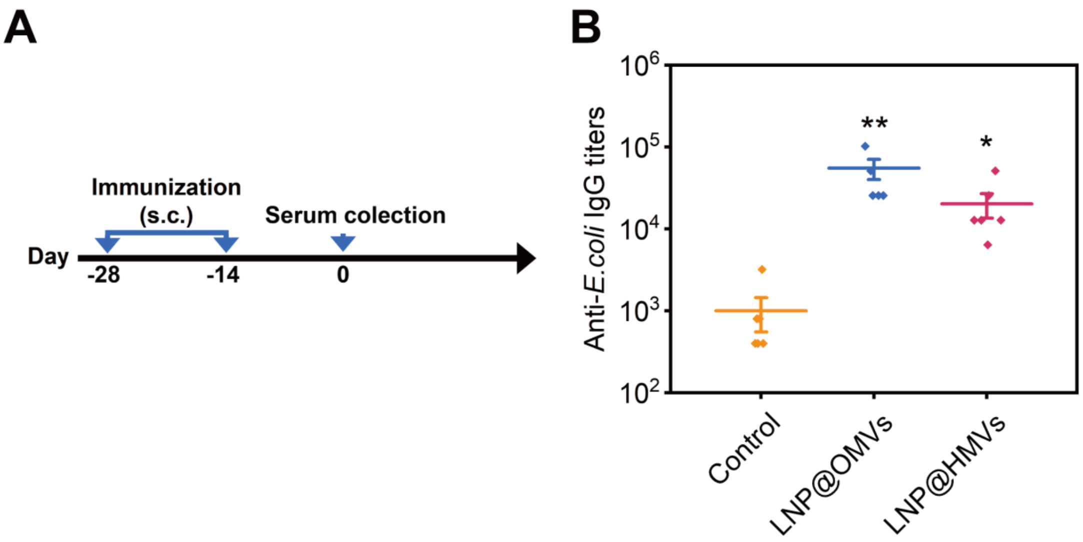

Fig. S15.

(**A**) Schematics of experimental design to evaluate the long-term *in vivo* immune responses. (**B**) Anti-*E. coli* specific antibody IgG titers at day 14 after the second immunization (n = 6). Naive mice without immunization were used as control. (*$p < 0.05$, **$p < 0.01$)

Table S1.

Preparation of LNPs at different weight ratios of glyceryl monostearate (GMS) and norfloxacin (Nor) (n = 3).

GMS : NOR	Size (nm)	PDI	Zeta potential (mV)	Encapsulation Efficiency (%)	Drug Loading (%)
-	159.68 ± 3.11	0.16 ± 0.01	-19.33 ± 5.36	-	-
5 : 1	134.12 ± 7.71	0.18 ± 0.03	-17.61 ± 2.57	19.42 ± 7.83	2.08 ± 0.46
6 : 1	132.74 ± 3.33	0.16 ± 0.03	-16.57 ± 2.72	25.25 ± 3.62	2.06 ± 0.29
7 : 1	130.76 ± 7.81	0.17 ± 0.01	-15.98 ± 0.73	33.46 ± 1.59	2.36 ± 0.14
10 : 1	141.17 ± 7.51	0.17 ± 0.03	-15.81 ± 0.86	37.46 ± 2.23	1.86 ± 0.10

Table S2.

Blood biochemical analysis (n=4).

Treatment	PBS	LNP-N@HMVs
ALT (U/L)	51.14 ± 4.32	47.41 ± 2.36（p=0.18）
AST (U/L)	166.66 ± 37.19	165.78 ± 15.11（p=0.97）
UREA (mM)	19.97 ± 1.94	21.02 ± 2.83（p=0.57）
CREA (μM)	25.33 ± 3.13	22.78 ± 2.01（p=0.22）
UA (μM)	292.06 ± 32.41	327.86 ± 66.98（p=0.37）
Ka (mM)	12.34 ± 0.41	13.05 ± 1.62（p=0.43）
Na (mM)	228.52 ± 34.58	236.04 ± 46.07（p=0.80）

Table S3.

Blood cell counts of mice treated with PBS or LNP-N@HMVs (n=5).

Treatment	PBS	LNP-N@HMVs
White blood cell count (10^9/L)	9.28 ± 2.38	9.04 ± 1.02（p=0.84）
Lymphocyte count (10^9/L)	6.84 ± 1.47	6.46 ± 1.02（p=0.65）
Monocyte count (10^9/L)	0.30 ± 0.10	0.26 ± 0.05（p=0.46）
Neutrophil count (10^9/L)	2.14 ± 0.82	2.32 ± 0.30（p=0.66）
Red blood cell count (10^{12}/L)	10.14 ± 0.77	9.71 ± 0.37（p=0.29）
Platelet count (10^9/L)	850.00 ± 198.69	877.2 ± 153.28（p=0.82）

Table S4.

Endotoxin content of OMVs (n=3).

Samples obtained from *E. coli*	Endotoxin (EU) / Protein (ng)	Endotoxin (ng) / Protein (ng)
Lysate	0.57 ± 0.02	0.11 ± 0.00
Outer Membrane	47.85 ± 6.27	9.59 ± 1.25
OMVs	2.20 ± 0.03	0.43 ± 0.01
